# Association of APOBEC mutagenesis with stromal and endothelial niche remodeling and PCDH9-linked signaling alterations in colorectal cancer

**DOI:** 10.3389/fimmu.2026.1835351

**Published:** 2026-07-16

**Authors:** Junting Chen, Jiaming Cao, Baosen Zhou, Lianshuang Zhao, Chang Zheng

**Affiliations:** 1 Department of Clinical Epidemiology and Evidence-based Medicine, The First Hospital of China Medical University, Shenyang, China; 2 Department of Laboratory Medicine, The First Hospital of China Medical University, Shenyang, China

**Keywords:** APOBEC mutagenesis, CRC, endothelial lineage reprogramming, PCDH9, tumor microenvironment remodeling

## Abstract

**Background:**

APOBEC cytidine deaminases generate characteristic TCW-context mutations that diversify cancer genomes, yet their functional impact on colorectal cancer (CRC) progression and the tumor microenvironment (TME) remains poorly defined.

**Methods:**

We integrated whole-exome sequencing, bulk and single-cell transcriptomics from multi-cohort datasets, supported by functional assays and *in vivo* xenograft models, to delineate the biological and clinical consequences of APOBEC activation in CRC. A machine-learning framework was used to derive an APOBEC activation–associated transcriptional signature (AAS).

**Results:**

Across multi-cohort datasets, AAS defined a colorectal cancer subtype with enriched TCW mutagenesis and significantly worse survival. High AAS tumors were associated with coordinated remodeling of the TME, including increased fibroblast and endothelial signatures and reduced cytotoxic immune infiltration. Single-cell analyses suggested that high AAS tumors were enriched for endothelial states with arterial and pro-angiogenic features. Conditioned-medium experiments further supported a potential tumor cell secretome-mediated link between APOBEC3B activation and endothelial transcriptional remodeling. Integrative analyses identified PCDH9 as a candidate APOBEC-associated target linked to Hippo, Wnt/β-catenin, and TGF-β signaling alterations, while mutation-specific causality remains to be experimentally validated.

**Conclusions:**

This multi-omics study identifies APOBEC activity as a central orchestrator of CRC evolution, associated with stromal-vascular remodeling and intracellular oncogenic activation through PCDH9-associated signaling alterations. The AAS provides a robust prognostic biomarker and highlights APOBEC-high tumors as candidates for anti-angiogenic therapy and for interventions targeting APOBEC-induced signaling vulnerabilities.

## 1 Introduction

Cancer develops through the progressive accumulation of somatic mutations, which generate genomic instability and enable deregulated cell growth (1, 2). The resulting mutational burden contributes to extensive phenotypic and genetic heterogeneity across tumors, complicating both disease classification and therapeutic intervention (3, 4). A subset of these alterations converges on driver genes, including oncogenes and tumor suppressors, whose persistent activation or loss provides essential fitness advantages for malignant cells (5). Classic examples such as EGFR mutations in lung cancer illustrate how tumors can become dependent on a single dominant driver pathway, creating clinically exploitable vulnerabilities (6). In colorectal cancer (CRC), recurrent mutations in KRAS, APC, and TP53 define major pathogenic routes (7–12). However, a substantial fraction of mutational events remains unexplained, and many putative drivers have yet to be functionally characterized.

Among endogenous mutational processes, the APOBEC family of cytidine deaminases has emerged as a major contributor to cancer genome diversification (13, 14). APOBEC enzymes catalyze cytosine-to-uracil deamination in single-stranded DNA, preferentially at TCW motifs, leading to characteristic C>T and C>G substitutions that manifest as COSMIC signatures SBS2 and SBS13 (15–19). Under inflammatory, viral, or therapeutic stress, aberrant APOBEC activation generates pervasive genomic lesions that accelerate tumor evolution and have been associated with metastasis, treatment resistance, and poor prognosis across multiple cancer types (20). Although APOBEC-related mutational patterns are detectable in CRC, how sustained APOBEC activity shapes downstream biological programs—beyond its role as a mutational source—remains largely undefined.

Emerging studies suggest that APOBEC dysregulation may influence tumor behavior through broader ecological effects on the tumor microenvironment (TME). For example, aberrant APOBEC3C expression in pancreatic cancer has been associated with characteristic genomic instability and altered immune infiltration (21). In addition, pan-cancer analyses have linked APOBEC/AID-related mutational processes with distinct tumor immune microenvironment patterns, suggesting that APOBEC-associated mutagenesis may interact with tumor ecological states in a context-dependent manner (21). Collectively, these observations raise the possibility that APOBEC-associated mutagenesis is linked to stromal, immune, or vascular remodeling. However, whether APOBEC activation contributes to TME reprogramming in CRC—and which cellular programs or APOBEC-targeted genes mediate such effects—has not been systematically characterized. Addressing this knowledge gap is essential for understanding how APOBEC-associated mutational processes interface with CRC progression and for identifying potential therapeutic vulnerabilities arising from these interactions.

In this study, we comprehensively characterize APOBEC-associated mutagenesis processes in CRC by integrating whole-exome sequencing (WES), bulk RNA sequencing, and single-cell transcriptomics with functional validation. We quantify APOBEC-mediated TCW mutagenesis, construct an APOBEC activation–associated transcriptional signature (AAS), and map its impact on stromal, immune, and endothelial cell states within the TME. Moreover, we identify candidate APOBEC-associated genes, notably PCDH9, and explore their potential mechanistic links to malignant progression. Together, these findings reveal APOBEC activity as a critical orchestrator of CRC evolution and highlight therapeutically actionable vulnerabilities arising from APOBEC-associated genetic and microenvironmental reprogramming.

## 2 Materials and methods

### 2.1 Patient samples and ethical approval

This study was approved by the Ethics Committee of the First Hospital of China Medical University (Approval No. [2025]001). We retrospectively collected clinical data and biological specimens from patients with primary CRC who underwent surgical resection between January 2018 and January 2022. Intraoperative fresh tumor tissues were obtained, immediately snap-frozen in liquid nitrogen or stored at −80 °C, and paired peripheral venous blood samples were collected prior to surgery. All samples were processed according to standardized protocols for subsequent WES and other molecular analyses. Clinicopathological characteristics and follow-up information were systematically curated, enabling correlation analyses and prognostic evaluation, including survival analysis and multivariable regression to identify independent prognostic factors.

### 2.2 Public datasets and data preprocessing

To increase statistical power and ensure generalizability, multi-omics and clinical data from external CRC cohorts were incorporated. WES, bulk RNA-seq, and clinical annotations for 546 CRC patients were retrieved from The Cancer Genome Atlas (TCGA-CRC). In addition, an independent MSK-CRC cohort from the study by Cercek et al. was included for external validation of APOBEC-associated mutational patterns. This cohort, available through cBioPortal under study Colorectal Cancer (MSK, JNCI 2021), contains targeted sequencing data generated using the MSK-IMPACT platform from 1,516 CRC samples, including 818 early-onset and 698 average-onset CRC cases with matched normal controls. Four independent GEO cohorts containing bulk transcriptomic data and survival information (GSE17536, n = 177; GSE17537, n = 55; GSE29621, n = 65; GSE39582, n = 566) were downloaded from the Gene Expression Omnibus. Two scRNA-seq datasets (GSE132465, n = 23; GSE144735, n = 12) were included to characterize cellular heterogeneity within the TME. Baseline characteristics for all cohorts are summarized in **Supplementary Tables 1**–**7**.

Bulk RNA-seq datasets were normalized and log-transformed as appropriate. For microarray datasets, probe-level intensities were mapped to gene symbols, and duplicate probes were averaged. Batch effects across validation cohorts were evaluated and corrected when necessary.

### 2.3 Whole-exome sequencing and somatic variant processing

The aim of WES processing was to obtain high-confidence somatic mutations for APOBEC-related trinucleotide analysis and downstream modeling. Genomic DNA extracted from tumor tissues and matched blood samples underwent library preparation using the Agilent SureSelect Human All Exon kit. Libraries passing quality control, including accurate insert size and sufficient effective concentration (>2 nM), were sequenced on the BGI platform with paired-end chemistry.

Reads were converted to FASTQ format and subjected to stringent quality filtering. High-quality reads were aligned to the hg19 reference genome using BWA-MEM, followed by PCR duplicate removal with Picard. Somatic variants were called using GATK Mutect2 following GATK Best Practices. Variants not meeting quality thresholds (QD ≤ 2, MQ ≤ 40, FS ≥ 60) were removed. Final variant calls were annotated using ANNOVAR.

Mutational signatures were extracted using the maftools R package. Somatic mutations were categorized into 96 trinucleotide contexts, and non-negative matrix factorization was applied to identify underlying mutational processes. The optimal signature number (k) was selected at the point of inflection in cophenetic correlation curves. Identified signatures were matched to COSMIC SBS signatures using cosine similarity.

### 2.4 Identification of TCW-mutated genes and APOBEC-associated candidates

To characterize genes potentially affected by APOBEC deaminase activity, somatic mutations derived from the WES pipeline were scanned for events occurring within the TCW motif (T[C→X]W, W = A/T). For each sample, mutations with the reference cytosine located within a TCW context were extracted, and genes harboring at least one TCW mutation were catalogued. A total of 5,309 TCW-mutated genes were identified and defined as APOBEC-associated candidates for downstream co-expression, machine-learning, and functional analyses.

### 2.5 Weighted gene co-expression network analysis

WGCNA was performed to identify gene modules associated with CRC progression and survival. Using the WGCNA R package, a series of candidate soft-thresholding powers was tested to approximate scale-free topology; β = 6 yielded the optimal network structure. Hierarchical clustering and dynamic tree-cutting were applied to detect co-expression modules. Module eigengenes were correlated with clinical traits, and modules significantly associated with tumor progression were selected. Genes from these modules were intersected with TCW-mutated genes to refine APOBEC-relevant candidates.

### 2.6 Machine learning–based construction of the APOBEC activation–associated signature

To derive a transcriptome-based signature associated with WES-defined APOBEC mutational activity and clinical outcome, we constructed the APOBEC activation-associated signature.

Feature selection. Univariate Cox regression was performed in TCGA-CRC to identify genes associated with overall survival. These genes were intersected with TCW-mutated genes and WGCNA-derived progression-related genes, producing a candidate gene set.

Model generation. Ten machine-learning algorithms—Random Survival Forest, Cox partial least squares (plsRcox), Elastic Net, CoxBoost, Lasso, Ridge, GBM, SuperPC, survival-SVM, and stepwise Cox—were combined into 100 unique algorithmic configurations. Models were trained and internally validated using leave-one-out cross-validation (LOOCV) in TCGA-CRC.

External validation. All models were evaluated across four independent GEO cohorts. Harrell’s concordance index (C-index) and its 95% confidence interval (CI) were computed for each model in every cohort. The model with the highest average C-index across all datasets was selected as the final AAS. No feature re-selection, model refitting, or cutoff optimization was performed in the external GEO validation cohorts.

### 2.7 Bulk RNA-seq library preparation and processing

Total RNA extracted from tumor tissues was assessed for purity, concentration, and integrity before library preparation. Poly(A)+ mRNA was enriched, fragmented, and reverse-transcribed to generate cDNA. After end-repair, A-tailing, adaptor ligation, and size selection, libraries were PCR-amplified and quantified. Libraries meeting quality thresholds were sequenced using paired-end 150 bp chemistry.

Raw reads were filtered to remove adaptor-contaminated sequences, reads with >10% ambiguous bases, or reads with >50% low-quality bases. Clean reads were aligned to the GRCh38 reference genome using HISAT2, and transcript assembly and quantification were performed using StringTie.

### 2.8 RT–qPCR validation of gene expression

Total RNA was isolated using RNAiso Plus (9109, Takara, Beijing, China), and cDNA was synthesized with a commercial reverse transcription kit. Quantitative PCR was performed using gene-specific primers (**Supplementary Table 8**) with GAPDH as the internal reference. Quantitative real-time PCR was subsequently performed to measure mRNA expression levels with the TB Green PCR Kit (RR820A, Takara) and a 7500 Fast Real-Time PCR System (Applied Biosystems). All experiments were repeated independently in triplicate.

### 2.9 Construction and evaluation of the prognostic nomogram

To construct a clinically interpretable prognostic nomogram, candidate variables included AAS, age, sex, tumor stage, T stage, N stage, and M stage. Univariate Cox regression was first performed to assess the association between each variable and overall survival. Variables with prognostic relevance were then entered into multivariate Cox regression to identify independent prognostic factors. To avoid overfitting and to maintain a parsimonious model, only variables that remained statistically significant in both univariate and multivariate Cox analyses and were sufficiently available across the analyzed cohorts were retained for final nomogram construction. Variables were not included in the final model when they were unavailable, incompletely annotated, or heterogeneously defined across public cohorts. The final nomogram was constructed using the rms and replot R packages, and its performance was evaluated by time-dependent ROC curves, calibration curves, and decision curve analysis.

### 2.10 Estimation of immune and stromal cell infiltration

To comprehensively characterize the composition of the TME in the TCGA-CRC cohort, immune and stromal cell infiltration was inferred using five complementary computational frameworks. EPIC was first applied to estimate the proportions of major immune and stromal lineages based on reference-based gene-expression deconvolution. CIBERSORT further quantified relative immune cell fractions through support vector regression using a predefined leukocyte signature matrix. To obtain absolute immune cell abundances, we employed quanTIseq, an RNA-seq–optimized deconvolution algorithm. MCP-counter was used to enumerate immune and stromal subsets based on robust marker gene signatures, while xCell provided an additional layer of resolution by integrating gene-set enrichment scores across 64 immune and non-immune cell types.

To minimize algorithm-specific biases and enhance the robustness of infiltration estimates, results from all methods were jointly evaluated and integrated, yielding consistent and reliable patterns of immune and stromal cell infiltration across the cohort.

### 2.11 Single-cell RNA-seq analysis

Raw scRNA-seq data were processed using Seurat. Cells with <300 or >10,000 detected genes, >20% mitochondrial transcripts, or >5% hemoglobin transcripts were removed. Data normalization, scaling, and identification of highly variable genes were performed. PCA was used for dimensionality reduction, FindClusters for clustering, and UMAP for visualization. Cell types were annotated using canonical marker genes.

### 2.12 Gene-set–based enrichment analyses (ssGSEA and GSVA)

To quantify the relative abundance of cell populations identified in scRNA-seq within bulk transcriptomes, ssGSEA was applied using GSVA. GSVA was further used to evaluate pathway-level biological differences across AAS-defined groups.

### 2.13 Cell–cell communication analysis

Cell–cell communication networks were inferred using CellChat. Overexpressed genes in each cell type were mapped to known ligand-receptor interactions. A probabilistic model estimated communication probability, enabling visualization of intercellular signaling alterations associated with APOBEC activation.

### 2.14 Trajectory inference of endothelial cell states

To infer endothelial cell (EC)-state transitions, differentiation trajectories of EC subtypes were reconstructed using Monocle2. Highly variable genes were selected for trajectory construction, and the DDRTree algorithm was used for nonlinear dimensionality reduction and pseudotime ordering. Differentially expressed genes across pseudotime and branch points (BEAM analysis) were used to identify regulators of endothelial state transitions. To address the limitations of Monocle2-based trajectory reconstruction, we further performed Monocle3 analysis. The Seurat object containing ECs was converted into a Monocle3 cell data set object, followed by preprocessing, dimensionality reduction, clustering, graph learning, and pseudotime ordering. In addition, we inferred cell-state transition directions using VECTOR. The method relies on the principle that the quantile polarization of a cell’s principal component values reflects its developmental hierarchy, allowing unsupervised identification of starting cells. Briefly, all UMAP dimensions of ECs were treated as an image and split into pixels. The largest connected pixel network was then created by linking adjacent pixels in UMAP space, and this network was used to determine the direction of cellular development.

### 2.15 Mfuzz clustering analysis

To characterize dynamic co-expression patterns, Mfuzz soft clustering was applied. The fuzzy c-means algorithm identified gene sets exhibiting similar expression trajectories, enabling detection of temporal or gradient-related expression changes with improved noise tolerance.

### 2.16 Enzyme-linked immunosorbent assay

Concentrations of human BMP-6 (HJ010, Epizyme) and WNT7B (CSB-EL026142HU, Cusabio) were measured using commercial sandwich ELISA kits according to the manufacturers’ instructions. Briefly, standards and samples were added to pre-coated microplates and incubated, followed by sequential incubation with biotinylated detection antibodies and streptavidin–HRP conjugates. After washing, TMB substrate was added, and absorbance at 450 nm was recorded. Sample concentrations were interpolated from standard curves generated in each assay.

### 2.17 Cell culture and plasmid transfection

HCT116 and LoVo CRC cell lines were obtained from the Stem Cell Bank of the Chinese Academy of Sciences (Shanghai, China) and maintained under recommended standard conditions. All cell lines were routinely tested and confirmed to be free of mycoplasma contamination and were passaged every 3 days to maintain exponential growth.

For overexpression experiments, logarithmically growing cells were transiently transfected with jetPRIME transfection reagent following the manufacturer’s protocol. Experimental groups comprised: (1) wild−type APOBEC3B overexpression (pcDNA3.1−A3B, OE-A3B); (2) catalytically inactive APOBEC3B overexpression (pcDNA3.1−A3B−mut, OE-A3B Mut); (3) combined wild−type APOBEC3B and Protocadherin 9 (PCDH9) overexpression (pcDNA3.1−A3B + pcDNA3.1−PCDH9, OE-A3B + PCDH9−OE);

### 2.18 Immunofluorescence staining

Tissue sections were permeabilized with 0.1% Triton X-100 for 10 min. After blocking with 5% bovine serum albumin for 1 h, the sections were incubated with primary antibodies (**Supplementary Table 9**) overnight at 4 °C. The next day, the sections were washed and then incubated with fluorophore-conjugated secondary antibodies at room temperature in the dark for 1 h. Cell nuclei were counterstained with DAPI for 5 min. The sections were mounted with antifade mounting medium, and images were acquired using a fluorescence microscope.

### 2.19 Western blotting

Cells were lysed in ice-cold RIPA buffer for 30 min and centrifuged to obtain protein supernatants. Protein concentrations were measured using the BCA assay. Equal amounts of protein were denatured in loading buffer, separated by SDS-PAGE, and transferred onto PVDF membranes. Membranes were blocked with 5% skim milk for 1 h at room temperature and incubated with primary antibodies (**Supplementary Table 9**) overnight at 4 °C. After washing with TBST, membranes were incubated with HRP-conjugated secondary antibodies for 1 h at room temperature. Bands were visualized using enhanced chemiluminescence (ECL).

### 2.20 Deaminase activity assay

Cytidine deaminase activity was quantified using a fluorescence-based assay. Total protein was extracted with lysis buffer (25 mM HEPES pH 7.9, 10% glycerol, 150 mM NaCl, 0.5% Triton X-100, 1 mM EDTA, 1 mM MgCl_2_, 1 mM ZnCl_2_, and protease inhibitors) and clarified by centrifugation at 15,000 g for 10 min at 4 °C. Cell lysates were incubated with a dual-labeled oligonucleotide probe, 5’-(6-FAM)-AAA-TTC-TAA-TAG-ATA-ATG-TGA-(TAMRA)-3’, at 37 °C for 2 h. In this probe, 6-FAM fluorescence is quenched by TAMRA via FRET. Deamination of cytidine to uridine within the probe allows subsequent treatment with 0.75 U uracil DNA glycosylase (UDG) at 37 °C for 45 min to generate abasic sites. Incubation with NaOH at 95 °C for 2 min cleaves the DNA at abasic positions, abolishing FRET and restoring 6-FAM fluorescence. Fluorescence (excitation/emission 490/520 nm) was measured with a microplate reader, and increased signal reflected deaminase activity.

### 2.21 Cell proliferation assay

Cell proliferation was assessed using the CCK-8 assay (Seven, SC119). Cells were seeded in 96-well plates at 2 × 10³ cells per well in 100 μL complete medium. At 0, 24, 48, and 72 h, 10 μL of CCK-8 solution was added to each well and incubated for 2 h at 37 °C in 5% CO_2_. Absorbance at 450 nm was recorded, and blank wells were used for background correction.

### 2.22 Cell apoptosis assay

Apoptosis was analyzed using Annexin V-APC/7-AAD staining (E-CK-A218, Elabscience). Cells were seeded in 6-well plates at 2 × 10^5^ cells per well, treated as indicated, and harvested. After two washes with cold PBS, cells were resuspended in 1× binding buffer, followed by sequential addition of 5 μL Annexin V-APC and 5 μL 7-AAD. After gentle mixing and 15 min incubation at room temperature in the dark, samples were analyzed by flow cytometry, and apoptotic fractions were quantified.

### 2.23 Transwell migration and invasion assays

Cell migration and invasion were evaluated using 8-μm pore Transwell inserts (Corning, 3422). For invasion assays, the upper chambers were pre-coated with Matrigel, and 2 × 10^5^ cells in serum-free medium were seeded into the upper chambers, while the lower chambers contained medium with 10% FBS as chemoattractant. Migration assays were performed similarly without Matrigel coating. After 24 h incubation at 37 °C with 5% CO_2_, cells on the upper surface of the membrane were removed with cotton swabs. Cells that had migrated or invaded to the lower surface were fixed, stained with crystal violet, imaged, and quantified in five random fields per insert using ImageJ.

### 2.24 *In vivo* subcutaneous xenograft model

Female BALB/c nude mice (5–6 weeks old) were purchased from Beijing Huafukang Biotechnology Co., Ltd. and maintained under specific pathogen-free (SPF) conditions (22 ± 2 °C, 50 ± 10% humidity, 12-h light/dark cycle). All procedures were approved by the Institutional Animal Care and Use Committee of China Medical University (Approval No. CMU20240905).

HCT116 cells (3 × 10^6^ in 100 μL PBS) stably transfected with A3B-OE or A3B-OE + PCDH9-OE constructs were subcutaneously injected into the right flank. Tumor growth was monitored every 3 days using a digital caliper. At predefined endpoints, mice were euthanized by gradual CO_2_ exposure in a closed chamber to minimize distress. Tumors were excised, measured, and processed for further analyses.

### 2.25 Statistical analysis

All statistical analyses were performed using R software (version 4.2.1) and appropriate R packages. Continuous variables were summarized as median (interquartile range) or mean ± SD, depending on distribution. Group comparisons were conducted using Wilcoxon rank-sum test or Student’s t-test for two groups, and Kruskal–Wallis test or one-way ANOVA for three or more groups, with Tukey’s *post-hoc* test when applicable. Categorical variables were compared using chi-square test or Fisher’s exact test. Correlations were evaluated using Pearson or Spearman coefficients as appropriate.

Survival analyses were performed using Kaplan–Meier curves with log-rank tests and Cox proportional hazards regression. Hazard ratios (HRs) and 95% confidence intervals (CIs) were reported. Multiple testing corrections were applied where required. All tests were two-sided, and p-values <0.05 were considered statistically significant (* p < 0.05, ** p < 0.01, *** p < 0.001, **** p < 0.0001).

## 3 Results

### 3.1 APOBEC-associated mutational enrichment defines a clinically relevant CRC subtype

**Figure 1:**
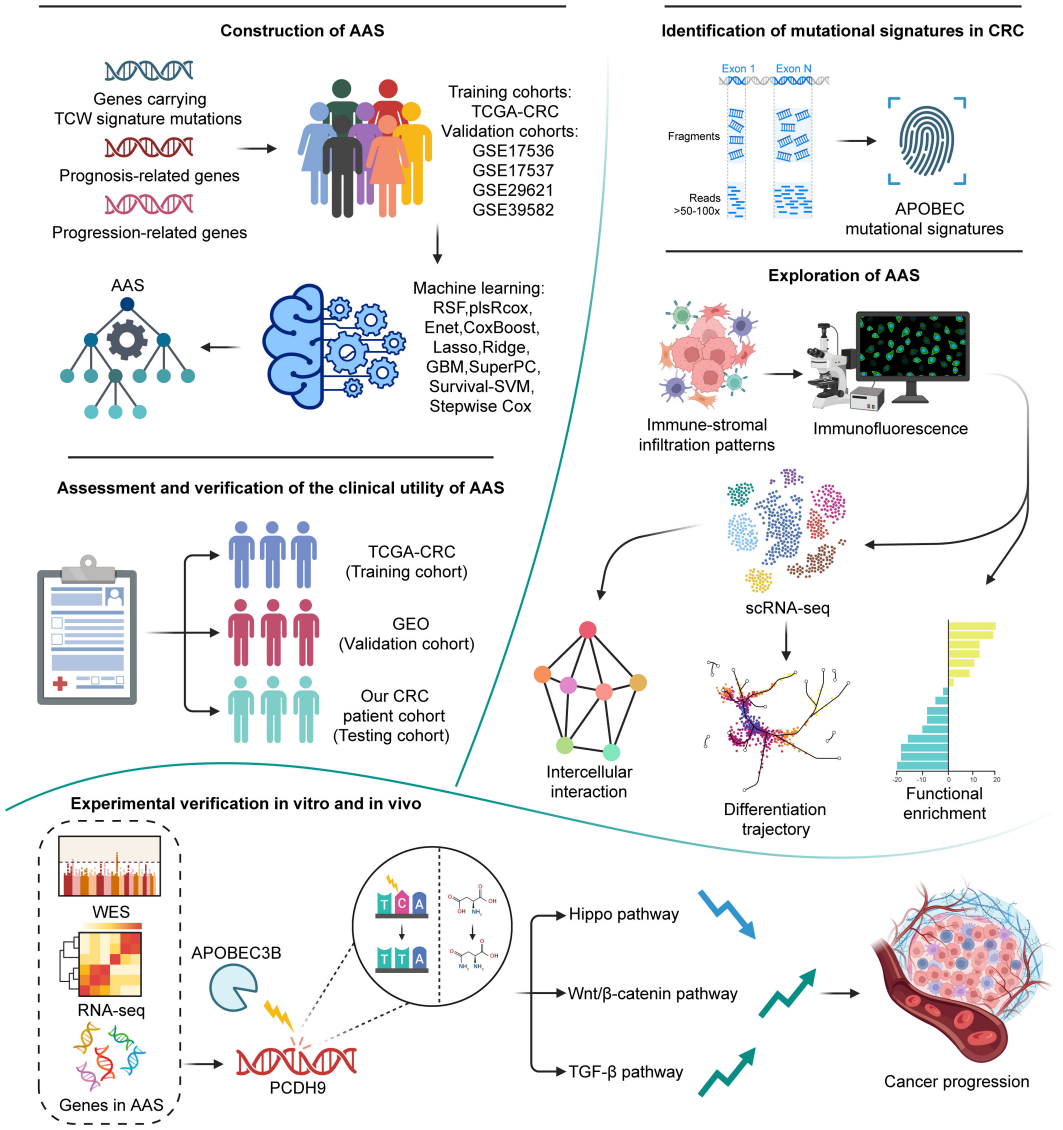
Schematic workflow of this study.

To determine whether APOBEC mutagenesis contributes to genomic diversification in CRC, we analyzed somatic mutation profiles from TCGA-CRC and an independent MSK-CRC mutation cohort. Because APOBEC enzymes preferentially deaminate cytosines within TCW trinucleotide motifs, generating C>T and C>G substitutions, we quantified these APOBEC-associated alterations (TCW mutations) in each patient as a surrogate measure of APOBEC activity. The overall study design and analytical workflow are summarized in **Figure 1**.

Across the TCGA-CRC cohort, TCW mutations varied widely among individuals, suggesting heterogeneity in APOBEC involvement. We next evaluated whether TCW mutation burden stratified clinical outcomes. Using log-rank statistics to identify the most discriminative cut-off, patients were separated into high- and low-TCW groups. Individuals with high TCW mutation enrichment exhibited significantly worse overall survival compared with those with low enrichment (**Supplementary Figure 1a**), indicating potential prognostic relevance.

To test whether this TCW-based grouping reflected bona fide APOBEC mutagenesis, we performed mutational signature decomposition. The high-TCW group showed strong enrichment of SBS13, the canonical APOBEC cytidine deaminase signature (C>G), whereas no APOBEC signatures were detectable in the low-TCW group (**Supplementary Figures 1b–e**). Analysis of the MSK-CRC cohort yielded highly consistent results: the high-TCW subgroup displayed both SBS2 and SBS13 signatures, while the low-TCW subgroup again lacked APOBEC features (**Supplementary Figures 1f–i**).

Together, these findings demonstrate that TCW mutational enrichment provides a robust readout of APOBEC activity in CRC. Importantly, APOBEC-associated mutagenesis predominantly occurs in the high-TCW subgroup and is linked to inferior clinical outcomes, suggesting that APOBEC-driven genomic instability may contribute to aggressive tumor behavior.

### 3.2 Construction of an APOBEC activity–associated prognostic signature

**Figure 2:**
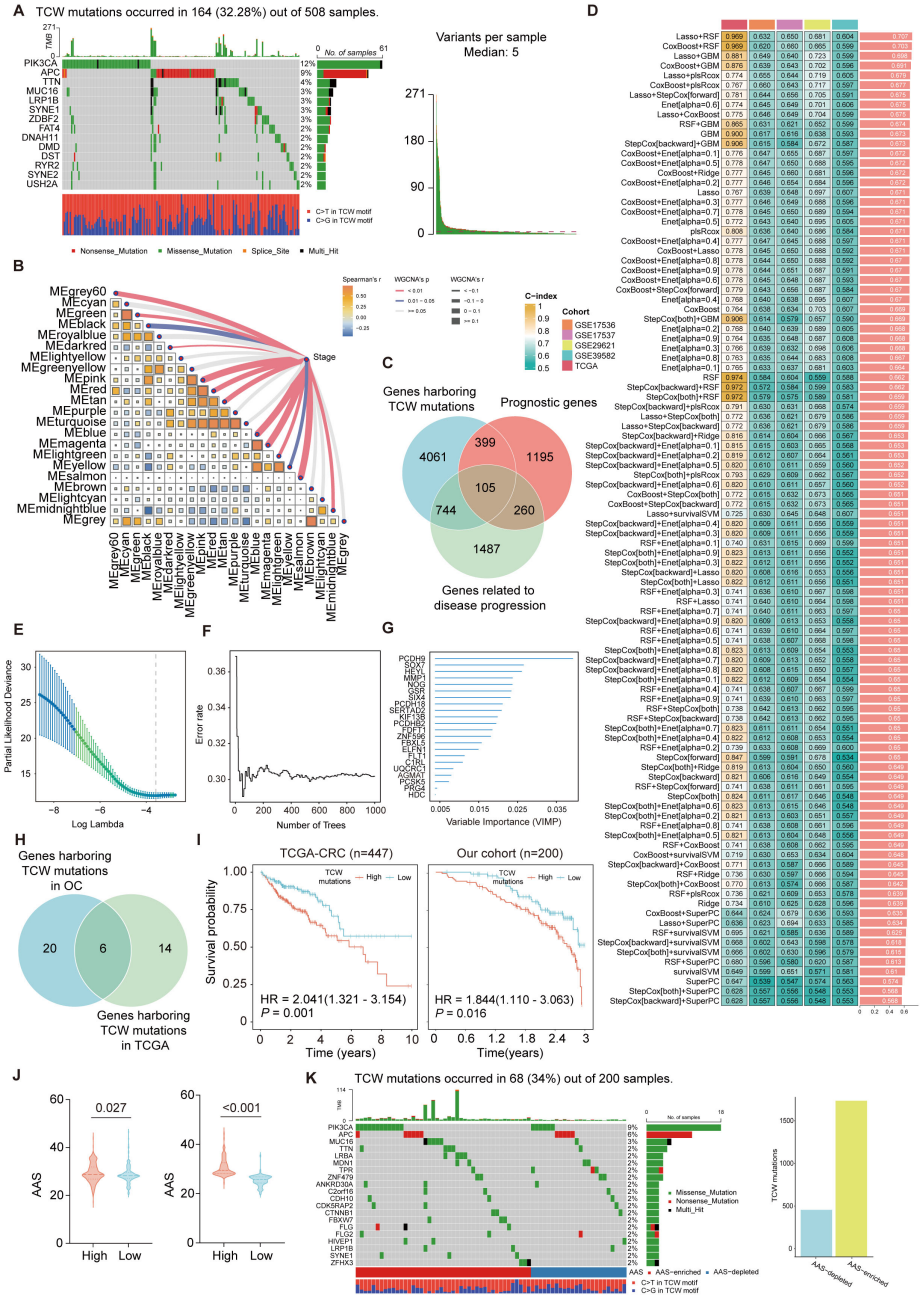
Identification of APOBEC-related key genes and construction of the prognostic signature. **(a)** Oncoplot showing the 14 genes with the highest frequencies of TCW mutations in the TCGA-CRC cohort. **(b)** Correlation heatmap displaying associations between clinical stage and WGCNA-derived gene modules. **(c)** Venn diagram illustrating the overlap among genes harboring TCW mutations, genes associated with disease progression, and prognosis-related genes. **(d)** Performance of 100 machine-learning models generated from 10 algorithms, ranked by the average C-index across four external validation cohorts; the Lasso–Random Survival Forest (RSF) model demonstrated the best predictive performance. **(e)** Partial likelihood deviance curve of the Lasso model across log-transformed λ values, with the vertical dashed line indicating the optimal λ. **(f)** Error rate plot for the RSF model showing progressive stabilization as the number of trees increases. **(g)** Variable-importance plot depicting the relative contribution of the 22 genes retained in the final prognostic signature. **(h)** Overlap of genes with high-frequency TCW mutations between the TCGA cohort and an independent validation cohort of 200 CRC samples. **(i)** Kaplan–Meier survival curves in TCGA-CRC (left) and the independent cohort (right), demonstrating significantly poorer outcomes in patients with high TCW mutation burden. **(j)** Violin plots comparing AAS scores between high- and low-TCW groups in TCGA-CRC (left) and the independent cohort (right). **(k)** Oncoplot showing the 20 most frequently TCW-mutated genes in the independent cohort (left) and comparison of TCW mutation counts between AAS-enriched and AAS-depleted tumors (right).

Having established that APOBEC mutational enrichment marks a biologically and clinically distinct CRC subtype, we next asked whether APOBEC genomic alterations translate into transcriptional programs with prognostic relevance. Across TCGA-CRC, we identified 5,309 genes harboring TCW mutations—the characteristic molecular footprint of APOBEC activity—with missense alterations representing the dominant variant class. The 14 most frequently mutated genes accounted for TCW mutations in approximately one-third of patients, indicating that APOBEC activity recurrently targets a non-random set of genes with potential functional consequences (**Figure 2a**).

To derive a transcriptional classifier that captures APOBEC-driven changes, we integrated multiple layers of evidence. Genes carrying TCW mutations were examined alongside those associated with tumor progression, as defined by WGCNA, and those linked to patient prognosis in univariate Cox analysis. The convergence of these datasets narrowed the list to 105 genes that not only bear APOBEC footprints but also exhibit dynamic changes along disease progression and measurable impact on survival (**Figures 2b, c**).

We then developed a multi-cohort machine-learning framework to determine which combination of these genes most robustly predicts outcome. Ten algorithms were systematically combined into 100 unique model configurations, and each model was rigorously evaluated through cross-validation in TCGA-CRC and external validation across four independent GEO cohorts. Among them, a model coupling Lasso-based feature refinement with the Random Survival Forest classifier consistently achieved the highest average concordance indices and was therefore selected as the optimal approach (**Figure 2d**). In the external GEO cohorts, its C-index values indicated moderate but reproducible prognostic discrimination. The corresponding 95% confidence intervals are now provided in **Supplementary Table 10**. The resulting AAS consists of 22 genes whose collective expression patterns reflect the transcriptional consequences of APOBEC mutagenesis (**Figures 2e–g**). Each patient receives an AAS score, providing a quantitative estimate of APOBEC-driven transcriptional activation.

To avoid conflating the transcriptome-based AAS with mutation-level APOBEC activity, we performed WES on an independent cohort of 200 CRC patients. Genes with high-frequency TCW mutations substantially overlapped with those from TCGA, confirming reproducible APOBEC targeting patterns across cohorts (**Figure 2h**). Patients with a higher TCW mutation burden showed significantly worse survival in both TCGA and the validation cohort (**Figure 2i**). Moreover, AAS scores were markedly elevated in tumors enriched for TCW mutations, and WES confirmed that AAS-high tumors harbor more APOBEC-associated mutations than AAS-low tumors (**Figures 2j, k**). These associations were reproduced in the independent WES validation cohort, supporting that AAS captures a transcriptional state associated with, but distinct from, mutation-defined APOBEC activity.

Together, these results support AAS as a biologically grounded APOBEC-associated transcriptional signature with moderate but reproducible prognostic performance across external cohorts.

### 3.3 Association of the APOBEC activation signature with clinical outcomes

**Figure 3:**
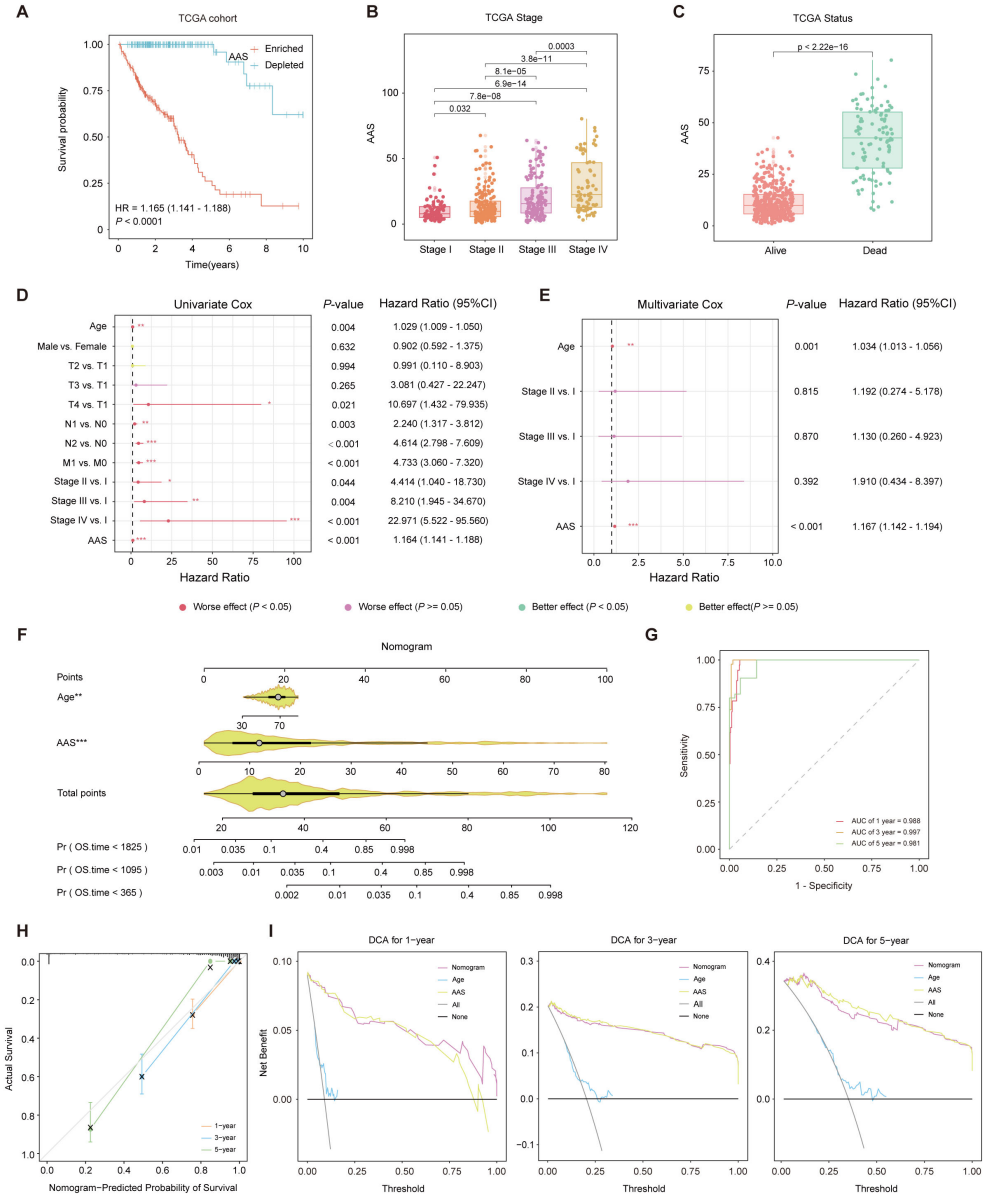
Evaluation of the prognostic value of AAS and construction of the clinical nomogram. **(a)** Kaplan–Meier survival curves comparing AAS-enriched and AAS-depleted patients in the TCGA-CRC cohort. **(b)** Distribution of AAS across clinical stages, showing progressive elevation with advancing disease. **(c)** Comparison of AAS between patients who were alive versus deceased, demonstrating significantly higher AAS among non-survivors. **(d)** Univariate Cox regression assessing clinicopathologic variables and AAS in TCGA-CRC. **(e)** Multivariate Cox regression confirming AAS and age as independent prognostic factors. **(f)** Nomogram integrating age and AAS to predict individualized 1-, 3-, and 5-year survival probabilities. **(g)** ROC curves evaluating the predictive accuracy of the nomogram at different time horizons. **(h)** Calibration curves comparing nomogram-predicted versus observed survival probabilities at 1, 3, and 5 years. **(i)** DCA demonstrating the superior net clinical benefit of the nomogram compared with individual clinical variables.

We next evaluated the clinical significance of the APOBEC AAS across multiple independent cohorts. Stratification of TCGA-CRC patients by median AAS demonstrated markedly inferior overall survival in the AAS-enriched group, a finding that remained consistent in the combined GEO datasets and in our independent cohort of 200 CRC cases (**Figure 3a**; **Supplementary Figures 2a, b**). Similar trends were observed across additional survival endpoints, including disease-free, disease-specific, and progression-free survival, underscoring the robustness of AAS as a survival discriminator (**Supplementary Figures 2c–e**). Although several datasets exhibited nonsignificant trends toward poorer outcomes in AAS-enriched tumors, these were largely attributable to limited sample sizes and reduced statistical power (**Supplementary Figures 2f–h**).

AAS also tracked with key clinical variables. Higher AAS values were observed in patients with advanced tumor stage and in those who had died or experienced progression, indicating that APOBEC-related transcriptional activation escalates alongside disease severity (**Figures 3b, c**; **Supplementary Figures 2i–m**). In univariate Cox regression, AAS emerged as a strong predictor of overall survival and retained its significance after adjustment for clinicopathologic factors in multivariate analysis, confirming its role as an independent prognostic determinant (**Figures 3d, e**).

To facilitate individualized survival estimation, we established a multivariable nomogram that integrates AAS with age. This nomogram showed high apparent time-dependent AUCs for 1-, 3-, and 5-year overall survival (**Figures 3f, g**). Calibration analyses demonstrated excellent concordance between predicted and observed outcomes (**Figure 3h**), and decision curve analysis (DCA) showed that the nomogram consistently outperformed conventional clinical variables in net clinical benefit across all time points (**Figure 3i**). To further evaluate generalizability, we performed the same analysis in the validation cohort. These findings were reproducible across validation cohorts (**Supplementary Figures 2n–r**, **3a–h**), demonstrating the stability and clinical utility of AAS-based prognostication.

Together, these results establish the AAS as an exploratory and independent prognostic indicator in CRC, linking APOBEC-driven transcriptional reprogramming to patient survival and providing a clinically actionable tool for risk stratification.

### 3.4 AAS-enriched tumors are associated with stromal enrichment and fibroblast expansion in CRC

**Figure 4:**
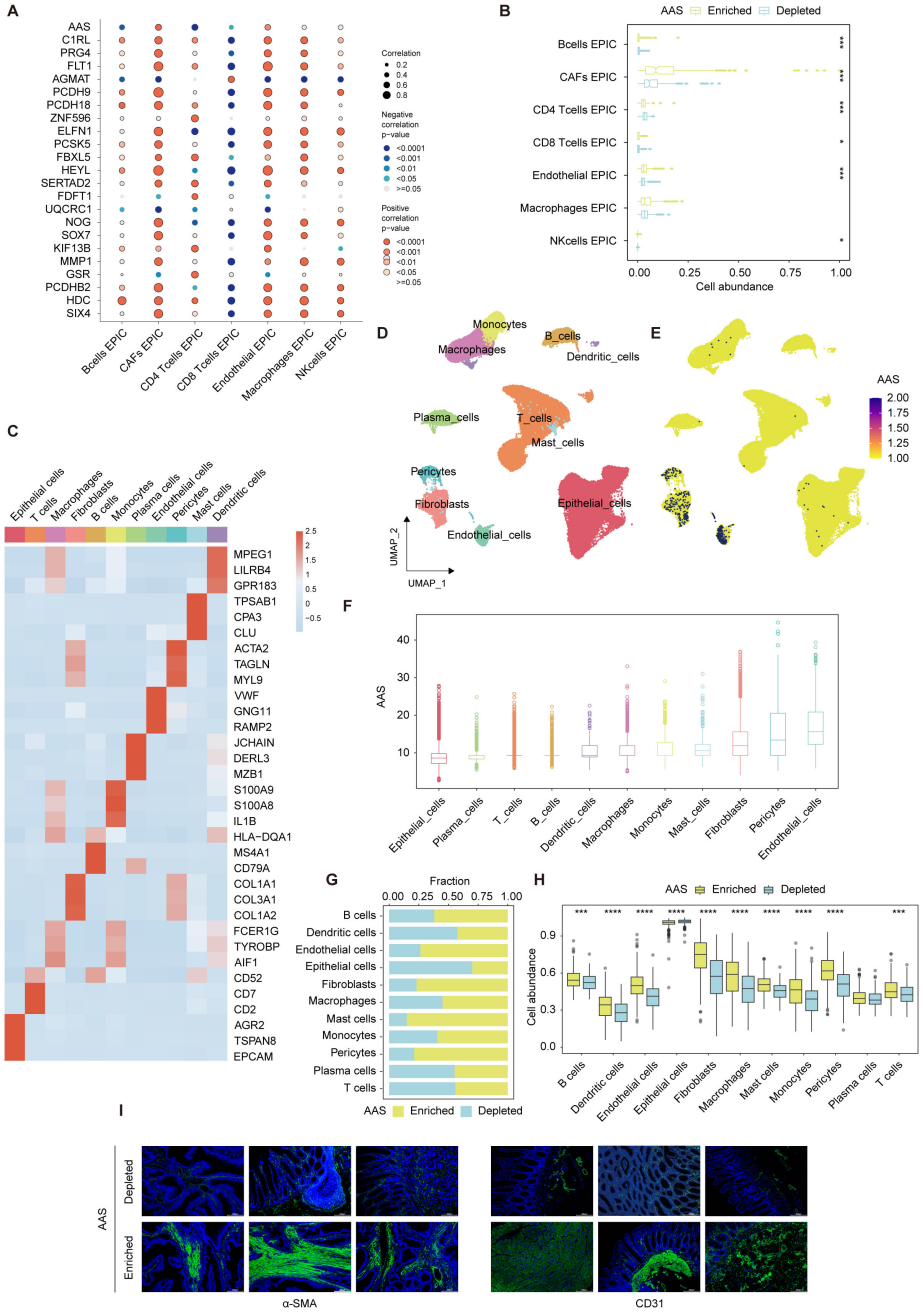
Association between cell infiltration and AAS at both bulk and single-cell transcriptomic levels. **(a)** Dot plot depicting correlations between AAS (and AAS model genes) and the abundance of multiple immune and stromal cell types estimated using the EPIC algorithm. **(b)** Boxplot comparing EPIC-estimated abundances of different cell types between AAS-enriched and AAS-depleted tumors. **(c)** Heatmap showing representative marker genes used to annotate major cell populations in the integrated scRNA-seq dataset. **(d)** UMAP visualization illustrating the clustering and annotation of the 11 identified cell populations. **(e)** UMAP visualization displaying the distribution of single-cell AAS scores across the TME. **(f)** Boxplot showing AAS distributions across the 11 major cell populations. **(g)** Stacked bar chart comparing the relative proportions of the 11 identified cell types between AAS-enriched and AAS-depleted tumors. **(h)** Boxplots showing TCGA bulk-estimated abundances of the 11 cell populations stratified by AAS groups. **(i)** Representative immunofluorescence images of α-SMA (fibroblasts) and CD31 (ECs) staining in tumor sections from AAS-enriched and AAS-depleted patients; nuclei are counterstained with DAPI. Scale bar = 200 μm. * p < 0.05, ** p < 0.01, *** p < 0.001, **** p < 0.0001.

To examine how APOBEC activity influences the cellular composition of the CRC TME, we assessed bulk and single-cell transcriptomic profiles across AAS-enriched and AAS-depleted tumors. Bulk deconvolution revealed that AAS-enriched tumors exhibited markedly elevated stromal infiltration, characterized by increased proportions of fibroblasts, ECs, and pericytes. In contrast, anti-tumor immune populations—including CD8^+^ T cells, dendritic cells, plasma cells, NK cells, and B cells—were significantly reduced (**Figures 4a, b**; **Supplementary Figures 4a–h**).

After quality control and integration of the two scRNA-seq datasets, 64,963 single cells from 35 CRC patients were retained for downstream analysis. Unsupervised clustering and marker-based annotation identified 11 major cell lineages within the TME: epithelial cells (22,509), T cells (21,027), monocytes (4,034), macrophages (3,938), fibroblasts (3,776), B cells (3,357), plasma cells (2,766), ECs (1,842), pericytes (1,207), dendritic cells (301), and mast cells (206). These data enabled high-resolution characterization of AAS-associated cellular-state patterns (**Figures 4c, d**). Among all cell types, fibroblasts exhibited the highest APOBEC activation levels, as evidenced by their markedly elevated single-cell AAS scores (**Figures 4e, f**) and their increased relative representation within AAS-enriched tumors (**Figure 4g**). This enrichment pattern was further validated in the TCGA cohort (**Figure 4h**).

Immunofluorescence staining validated these transcriptomic observations: tumors with high AAS exhibited markedly increased α-SMA^+^ fibroblast density compared with AAS-depleted tumors (**Figure 4i**). To evaluate whether fibroblast expansion reflected lineage remodeling rather than recruitment, we performed pseudotime analysis. Fibroblasts displayed limited transcriptional heterogeneity and lacked distinct differentiation trajectories, indicating that APOBEC activation primarily amplifies fibroblast abundance rather than inducing novel fibroblast states (**Supplementary Figure 5**).

Together, these findings suggest that APOBEC activation is associated with profound remodeling of the TME, characterized by enhanced stromal dominance—particularly fibroblasts—and suppressed anti-tumor immunity, and this remodeling correlates with a microenvironmental state conducive to tumor progression.

### 3.5 AAS-enriched tumors display enhanced stromal-immune communication and pro-tumor signaling networks

**Figure 5:**
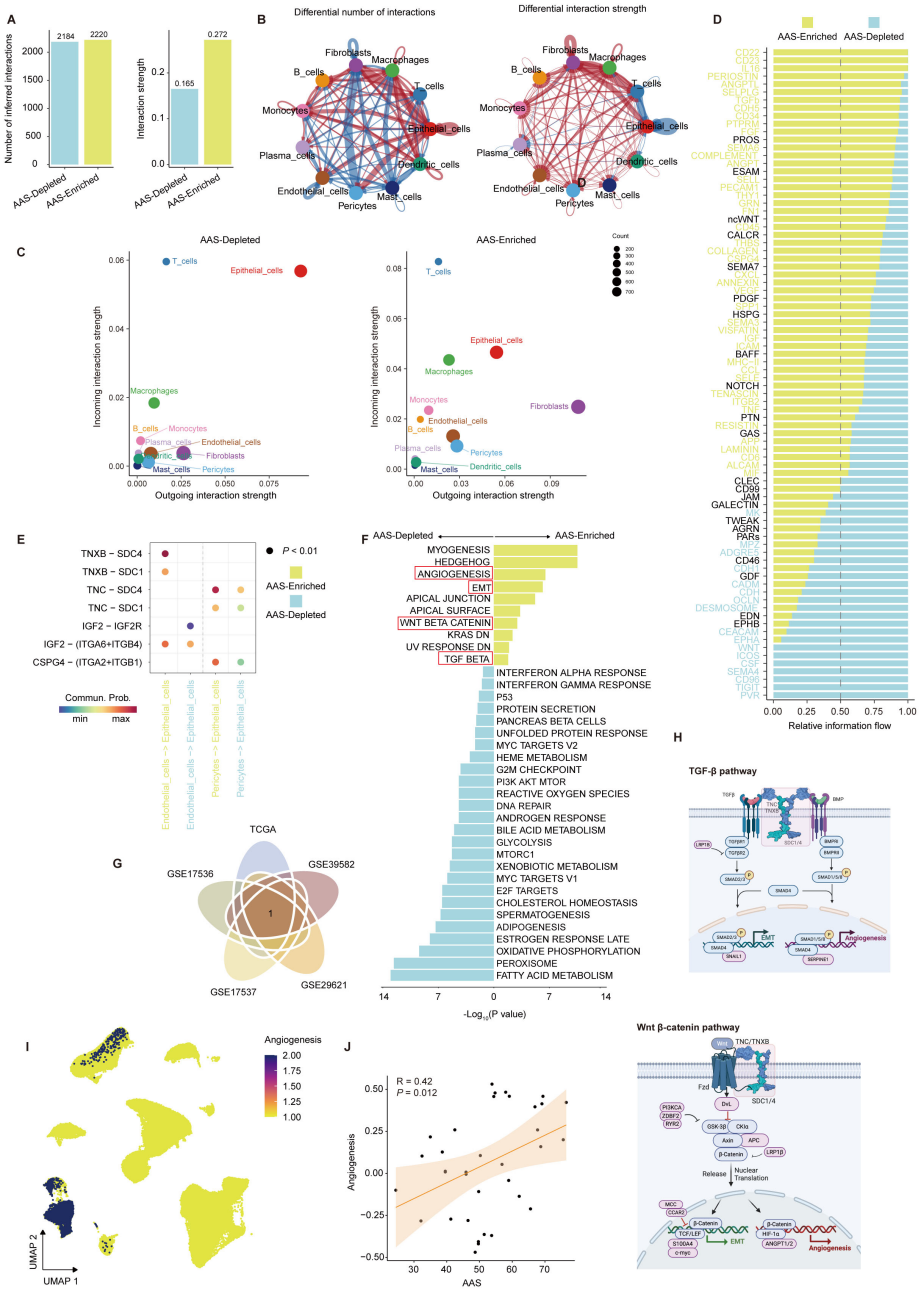
Comprehensive analysis of cell–cell communication and associated biological functions in AAS-enriched and AAS-depleted tumors. **(a)** Bar plots showing the total number of inferred cell–cell interactions (left) and the overall interaction strength (right) in AAS-enriched and AAS-depleted groups. **(b)** Circle plots comparing the number (left) and strength (right) of cell–cell interactions among TME lineages between AAS-enriched and AAS-depleted tumors. Red indicates increased interaction number or strength in AAS-enriched tumors, whereas blue indicates the opposite. **(c)** Bubble plots depicting outgoing and incoming interaction strengths for each major cell type in AAS-enriched (right) and AAS-depleted (left) groups. **(d)** Bar plot ranking signaling pathways according to their relative information flow in AAS-enriched and AAS-depleted tumors. **(e)** Bubble plot illustrating significantly altered ligand–receptor pairs mediating communication between specific sender and receiver cell types in AAS-enriched and AAS-depleted groups. **(f)** Bar plot showing biological functions significantly enriched in AAS-enriched (red) and AAS-depleted (blue) tumors. **(g)** Venn diagram demonstrating the overlap of significantly enriched biological functions identified across five independent cohorts (TCGA, GSE17536, GSE17537, GSE29621, and GSE39582). **(h)** Schematic illustration summarizing APOBEC-associated activation of TGF-β (top) and Wnt/β-catenin (bottom) signaling pathways. The diagram depicts representative ligand–receptor interactions and downstream signaling components involved in EMT and angiogenesis. **(i)** UMAP plot visualizing the distribution of angiogenesis activity at single-cell resolution. **(j)** Scatter plot showing the positive correlation between AAS and angiogenesis activity across samples.

To investigate how APOBEC-associated stromal expansion alters intercellular communication, we applied CellChat to construct signaling networks across 11 TME lineages. AAS-enriched tumors showed a marked increase in both the number and overall strength of cell–cell interactions, indicating a globally heightened communication landscape (**Figure 5a**). Comparison of communication networks revealed that AAS-enriched tumors exhibited stronger and more frequent interactions among stromal and immune-suppressive lineages, particularly involving fibroblasts, ECs, and pericytes (**Figures 5b, c**). Pathway-level analysis revealed that AAS enrichment preferentially amplified signaling programs associated with immune suppression, chronic inflammation, extracellular matrix remodeling, and angiogenesis, whereas AAS-depleted tumors favored pathways linked to immune activation and epithelial structural integrity (**Figure 5d**).

At the ligand–receptor level, stromal cells—particularly fibroblasts, ECs, and pericytes—served as major signal senders, displaying strengthened interactions involving ECM-associated molecules such as tenascin, FN1, THBS, CSPG4, and collagen, which more prominently engaged epithelial receptors in AAS-enriched tumors (**Figure 5e**; **Supplementary Figures 6a, b**). In contrast, WNT ligand–receptor interactions were relatively more active in AAS-depleted tumors, consistent with a more epithelial-preserving signaling environment (**Supplementary Figure 6a**). To further contextualize these findings, GSVA across five independent cohorts revealed consistent enrichment of fibroblast-associated pathways—including epithelial–mesenchymal transition and TGF-β signaling—in AAS-enriched tumors (**Figures 5f, g**; **Supplementary Figures 6c–f**). A mechanistic illustration of APOBEC-associated activation of Wnt/β-catenin and TGF-β signaling is provided in **Figure 5h**. Given the prominent involvement of vascular signaling (**Figure 5g**), we quantified angiogenesis activity across single-cell datasets. Angiogenic programs were elevated across ECs, fibroblasts, pericytes, monocytes, and macrophages within AAS-enriched tumors (**Figure 5i**), and AAS strongly correlated with angiogenic activity at the sample level (**Figure 5j**).

Collectively, these results indicate that APOBEC activation correlates with enhanced pro-tumor intercellular communication and is associated with alterations in key stromal, immune, and vascular signaling circuits, which together point toward a highly interactive and immunosuppressive TME.

### 3.6 AAS-enriched tumors are associated with endothelial state shifts toward arterial and pro-angiogenic phenotypes

**Figure 6:**
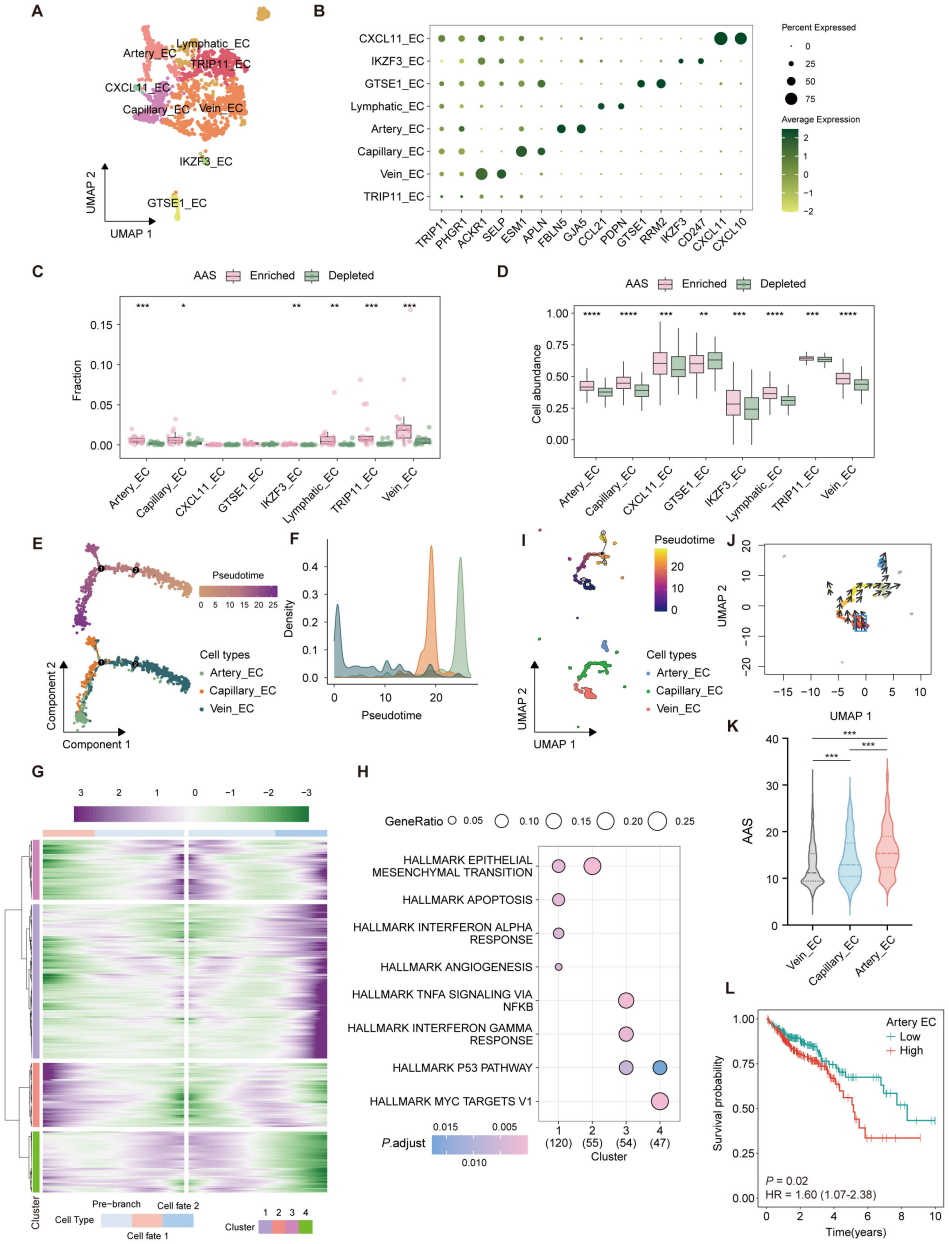
Single-cell characterization of endothelial cell heterogeneity, developmental dynamics, and prognostic relevance in CRC. **(a)** UMAP visualization of endothelial cells (ECs) reveals eight transcriptionally distinct subpopulations, including Ven-EC, Cap-EC, Art-EC, lymphatic EC, and four stress-associated subsets (CXCL11^+^ EC, GTSE1^+^ EC, IKZF3^+^ EC, and TRIP11^+^ EC). **(b)** Dot plot showing the expression patterns of representative marker genes across the eight endothelial subtypes, confirming their molecular identities. **(c)** Proportions of endothelial subtypes derived from scRNA-seq data comparing AAS-enriched and AAS-depleted tumors. **(d)** Estimated abundance of endothelial subtypes inferred from TCGA-CRC bulk RNA-seq, demonstrating consistent enrichment patterns across datasets. **(e)** Monocle2 pseudotime trajectory analysis illustrating the inferred developmental continuum of ECs. **(f)** Density distributions of major EC lineages (Ven-EC, Cap-EC, Art-EC) along pseudotime, highlighting their sequential progression during endothelial maturation. **(g)** Heatmap depicting dynamic gene expression changes across pseudotime, illustrating transcriptional programs associated with distinct stages of endothelial differentiation. **(h)** Functional enrichment analysis of four gene clusters identified along the pseudotime trajectory, revealing stage-specific activation of EMT, angiogenesis, interferon signaling, TNF-α/NF-κB, apoptosis, p53, MYC targets, and other hallmark pathways. **(i)** Monocle3 pseudotime trajectory analysis illustrating the inferred developmental continuum of ECs. **(j)** VECTOR direction on UMAP. **(k)** Violin plot comparing AAS levels among Ven-EC, Cap-EC, and Art-EC, showing a progressive increase along the venous-to-arterial axis. **(l)** Kaplan–Meier curves demonstrating that patients with high arterial EC abundance exhibit significantly poorer overall survival. * p < 0.05, ** p < 0.01, *** p < 0.001, **** p < 0.0001.

Given the markedly enhanced angiogenic signaling observed in AAS-enriched tumors, we next examined how APOBEC activation reshapes EC heterogeneity. Single-cell profiling identified eight transcriptionally distinct EC populations—venous EC (Ven-EC), capillary EC (Cap-EC), arterial EC (Art-EC), lymphatic EC, and four stress-responsive subsets (CXCL11^+^, GTSE1^+^, IKZF3^+^, and TRIP11^+^ ECs)—which together captured the vascular diversity of CRC (**Figures 6a, b**). Multiple EC subsets, including Ven-EC, Cap-EC, Art-EC, lymphatic EC, IKZF3^+^ EC, and TRIP11^+^ EC, were consistently expanded in AAS-enriched tumors across both single-cell and bulk transcriptomic datasets (**Figures 6c, d**), indicating broad endothelial remodeling under APOBEC activation.

Trajectory inference revealed a transcriptional continuum along a Ven-EC → Cap-EC → Art-EC axis (**Figures 6e, f**). Gene program dynamics along pseudotime indicated that early venous states were dominated by inflammatory and stress-response signatures, intermediate capillary states by the emergence of epithelial–mesenchymal transition features, and late arterial states by enhanced angiogenesis, apoptosis, interferon responses, and further EMT activation (**Figures 6g, h**). To further evaluate the directionality of endothelial state transitions and to address the limitations of Monocle2-based pseudotime inference, we performed additional Monocle3 and vector-based directional analyses. Consistent with the original Monocle2 results, Monocle3 and VECTOR-based directional analysis provided an additional layer of support for this inferred state transition (**Figures 6i, j**). Notably, AAS increased monotonically along this venous-to-arterial continuum (**Figure 6k**), potentially linking APOBEC activity to progressive endothelial maturation and the acquisition of arterialized, pro-angiogenic phenotypes. Consistent with their functional relevance, high Art-EC abundance was associated with significantly worse overall survival (HR = 1.60; 95% CI 1.07–2.38) in the TCGA-CRC cohort (**Figure 6l**).

To experimentally validate APOBEC-directed endothelial reprogramming, ECs were stimulated with supernatant from OE-A3B CRC cells and profiled over time using bulk RNA sequencing. Mfuzz clustering recapitulated the pseudotime-defined sequence of endothelial maturation, showing early induction of venous markers, a transitional rise in capillary signatures, and delayed activation of arterial markers (**Supplementary Figure 7a**). Translational analyses further supported the clinical relevance of these findings: AAS-depleted patients receiving anti-angiogenic therapy showed superior outcomes compared with AAS-enriched patients (**Supplementary Figure 7b**), and AAS-enriched tumors exhibited higher expression of VEGFR, FGFR, and PDGFR-α (**Supplementary Figures 7c, d**), indicative of heightened vascular signaling dependency.

Together, these findings are consistent with a potential link between APOBEC activation and endothelial lineage reprogramming and arterial fate commitment, which may contribute to a highly angiogenic vascular state in tumors that is associated with aggressive clinical behavior.

### 3.7 APOBEC3B activation is accompanied by TCW-context mutations and altered secretome-related factors

APOBEC3B expression was significantly higher than APOBEC3A expression in the TCGA cohort (**Supplementary Figure 8a**). In addition, random forest analysis showed that APOBEC3B had a greater contribution to TCW mutation burden than APOBEC3A, as indicated by higher variable importance scores (**Supplementary Figure 8b**). These findings support APOBEC3B as the more relevant APOBEC family member in the analyzed CRC cohorts and provide a rationale for using an APOBEC3B-focused experimental model. Therefore, we focused on APOBEC3B in subsequent experimental validation.

To address whether APOBEC3B activation could induce progressive TCW-context mutational accumulation over time, we established a doxycycline-inducible Tet-On APOBEC3B model and performed WES at weeks 0, 4, 8, and 12. Four experimental conditions were included: Tet-On A3B + Dox, Tet-On A3B − Dox, empty vector + Dox, and a catalytically dead A3B mutant. At baseline, all groups showed comparable low numbers of TCW-context mutations. After doxycycline induction, the Tet-On A3B + Dox group exhibited a marked and time-dependent increase in TCW mutations, whereas the Tet-On A3B − Dox, empty vector + Dox, and catalytic-dead A3B mutant groups showed only low-level background accumulation over time. The difference between the Tet-On A3B + Dox group and the three control groups became evident at week 4 and further increased at weeks 8 and 12 (**Supplementary Figure 8c**). These findings indicate that sustained APOBEC3B induction is sufficient to generate progressive TCW-context mutational accumulation in a catalytic-activity-dependent manner. Compared with transient APOBEC3B overexpression, this inducible system provides a more temporally resolved model for investigating APOBEC3B-associated mutational consequences, although it remains an experimental approximation of endogenous APOBEC evolution in human tumors.

**Figure 7:**
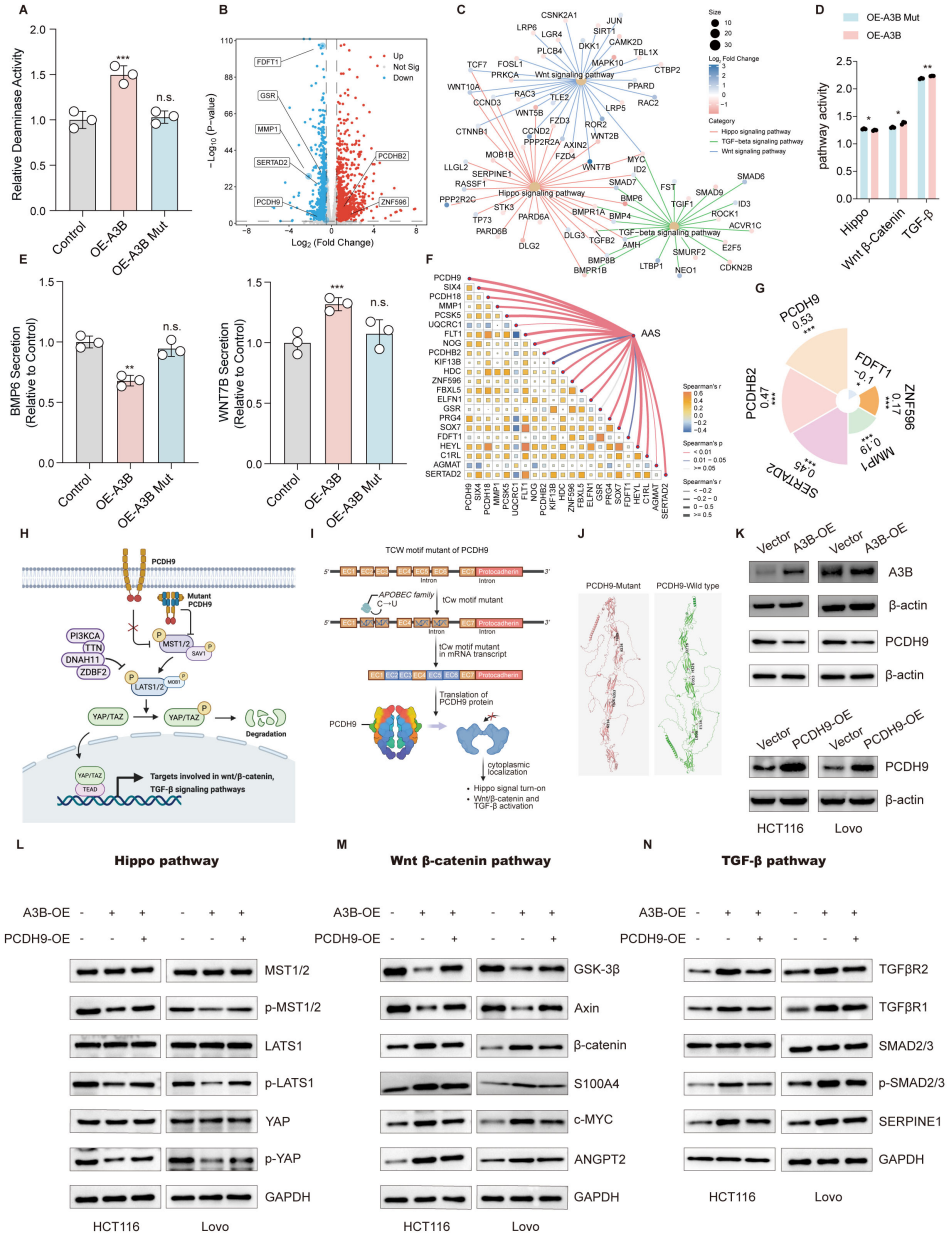
PCDH9 as a candidate effector linked to APOBEC-associated pro-tumorigenic signaling in CRC. **(a)** Relative deaminase activity measured in control, OE-APOBEC3B Mut, and OE-APOBEC3B CRC cells. **(b)** Volcano plot showing differentially expressed genes between OE-A3B and OE-A3B Mut groups. **(c)** Network visualization of pathway enrichment results highlighting significant involvement of Hippo, Wnt/β-catenin, and TGF-β signaling pathways. **(d)** Bar plot comparing activity scores of Hippo, Wnt/β-catenin, and TGF-β pathways between OE-A3B Mut and OE-A3B groups. **(e)** ELISA quantification of BMP6 (left) and WNT7B (right) secretion in the indicated cell groups. **(f)** Correlation heatmap illustrating associations between the 22 AAS signature genes and the APOBEC AAS. **(g)** Polar bar plot showing correlation coefficients of six top AAS-associated genes. **(h)** Schematic diagram showing the proposed working model in which APOBEC-associated alteration of PCDH9 may contribute to Hippo, Wnt/β-catenin, and TGF-β signaling changes. **(i)** Schematic representation of TCW motif mutations identified in PCDH9 and their predicted structural impact. **(j)** Predicted 3-D structures of APOBEC-associated mutant PCDH9 protein (left) and wild-type (right), suggesting a potential conformational impact of the mutation. These structural predictions were used to generate mechanistic hypotheses and do not constitute direct experimental evidence of altered protein stability or function. **(k)** Western blot validation of APOBEC3B and PCDH9 overexpression in HCT116 and LoVo cells. **(l)** Western blot analysis of Hippo pathway components (MST1/2, p-MST1/2, LATS1, p-LATS1, YAP, p-YAP) under indicated conditions. **(m)** Western blot analysis of Wnt/β-catenin pathway proteins (GSK-3β, Axin, β-catenin, S100A4, c-MYC, ANGPT2). **(n)** Western blot analysis of TGF-β pathway components (TGFβR1, TGFβR2, SMAD2/3, p-SMAD2/3, SERPINE1). * p < 0.05, ** p < 0.01, *** p < 0.001, **** p < 0.0001.

To investigate the molecular consequences of APOBEC activity in CRC, we established an OE-APOBEC3B cell model, which showed markedly elevated cytidine deaminase activity (**Figure 7a**). Whole-exome sequencing revealed widespread TCW-context mutations, consistent with APOBEC-associated mutagenesis. Among genes with the highest TCW mutation burden, 29% and 43% overlapped with recurrently mutated genes in our CRC cohort and TCGA-CRC, respectively (**Supplementary Figure 8d**), validating the biological relevance of our APOBEC3B-associated model.

Transcriptomic profiling of the cell model was used to evaluate whether the patient-derived AAS contained a tumor-cell-intrinsic component responsive to APOBEC3B. Among the 22 AAS genes, 7 showed statistically significant differential expression between OE-A3B and OE-A3B Mut cells (**Figure 7b**; **Supplementary Figure 8e**). We interpret this finding as partial recapitulation of a cell-autonomous subset of the AAS rather than complete reproduction of the patient-derived signature. This distinction is important because the AAS was derived from bulk patient tumors and therefore reflects not only tumor-cell-intrinsic APOBEC-associated transcriptional changes, but also stromal, immune, vascular, and long-term evolutionary components that cannot be fully modeled in a single *in vitro* CRC cell system. Functional enrichment analysis of AAS genes highlighted Hippo, Wnt/β-catenin, and TGF-β related pathways, suggesting that a subset of the AAS may be connected to tumor cell intrinsic signaling alterations induced by APOBEC3B (**Figures 7c, d**; **Supplementary Figure 8f**). Therefore, the APOBEC3B model was used to explore mechanistic candidates within the AAS, rather than to validate the entire 22 genes signature.

Because many APOBEC-associated genes encode secreted or membrane-associated factors, we next assessed whether APOBEC alters the tumor secretome. ELISA confirmed that OE-A3B cells secreted significantly higher levels of WNT7B but lower levels of BMP6 compared with controls (**Figure 7e**). These reciprocal changes in key morphogens suggest that APOBEC-linked mutagenesis contributes not only to intracellular pathway alteration but also to remodeling of paracrine signaling within the TME.

Correlation analysis further demonstrated that a subset of APOBEC-associated genes tracked with the AAS in patient tumors (**Figure 7f**). Among them, PCDH9 showed the highest correlation and ranked as the top hub gene in our AAS model (**Figure 7g**; **Supplementary Figure 8g**), highlighting it as a prime candidate for mediating the functional consequences of APOBEC-induced mutational injury.

### 3.8 PCDH9 restoration attenuates APOBEC3B-associated oncogenic signaling and malignant phenotypes

Given its strong association with the AAS and its established role as a membrane-associated tumor suppressor, we investigated whether PCDH9 serves as a candidate functional effector within the APOBEC-associated signaling program. A mechanistic illustration (**Figure 7h**) summarizes our working model: APOBEC-induced mutations impair PCDH9 function, thereby inactivating the Hippo pathway and releasing downstream constraints on Wnt/β-catenin and TGF-β signaling.

Consistent with this model, APOBEC3B-associated TCW-mutations were detected within the cadherin repeat domain of PCDH9 (**Figure 7i**). Structural modeling predicted that this mutation may disrupt the native fold of the PCDH9 extracellular domain (**Figure 7j**), suggesting a potential conformational alteration.

To experimentally verify pathway perturbations, we first confirmed the successful overexpression of APOBEC3B and PCDH9 in CRC cell lines by Western blotting (**Figure 7k**). After confirming successful overexpression, we next examined pathway-level effects. OE-A3B cells exhibited marked suppression of Hippo signaling, as indicated by reduced phosphorylation of MST1/2, LATS1, and YAP (**Figure 7l**). In parallel, APOBEC3B overexpression led to hyperactivation of Wnt/β-catenin signaling, with decreased GSK-3β and Axin levels and increased β-catenin accumulation, along with elevated expression of pro-oncogenic factors S100A4, c-MYC, and ANGPT2 (**Figure 7m**). The TGF-β pathway was also activated, as shown by increased levels of TGFβR1, TGFβR2, p-SMAD2/3, and SERPINE1 (**Figure 7n**). Importantly, restoring PCDH9 expression in OE-A3B cells reversed these pathway alterations, reinstating Hippo signaling and suppressing Wnt/β-catenin and TGF-β activation. These data support PCDH9 as a candidate APOBEC-associated effector linked to Hippo, Wnt/β-catenin, and TGF-β signaling changes, while not excluding the contribution of additional APOBEC3B targets acting in parallel.

**Figure 8:**
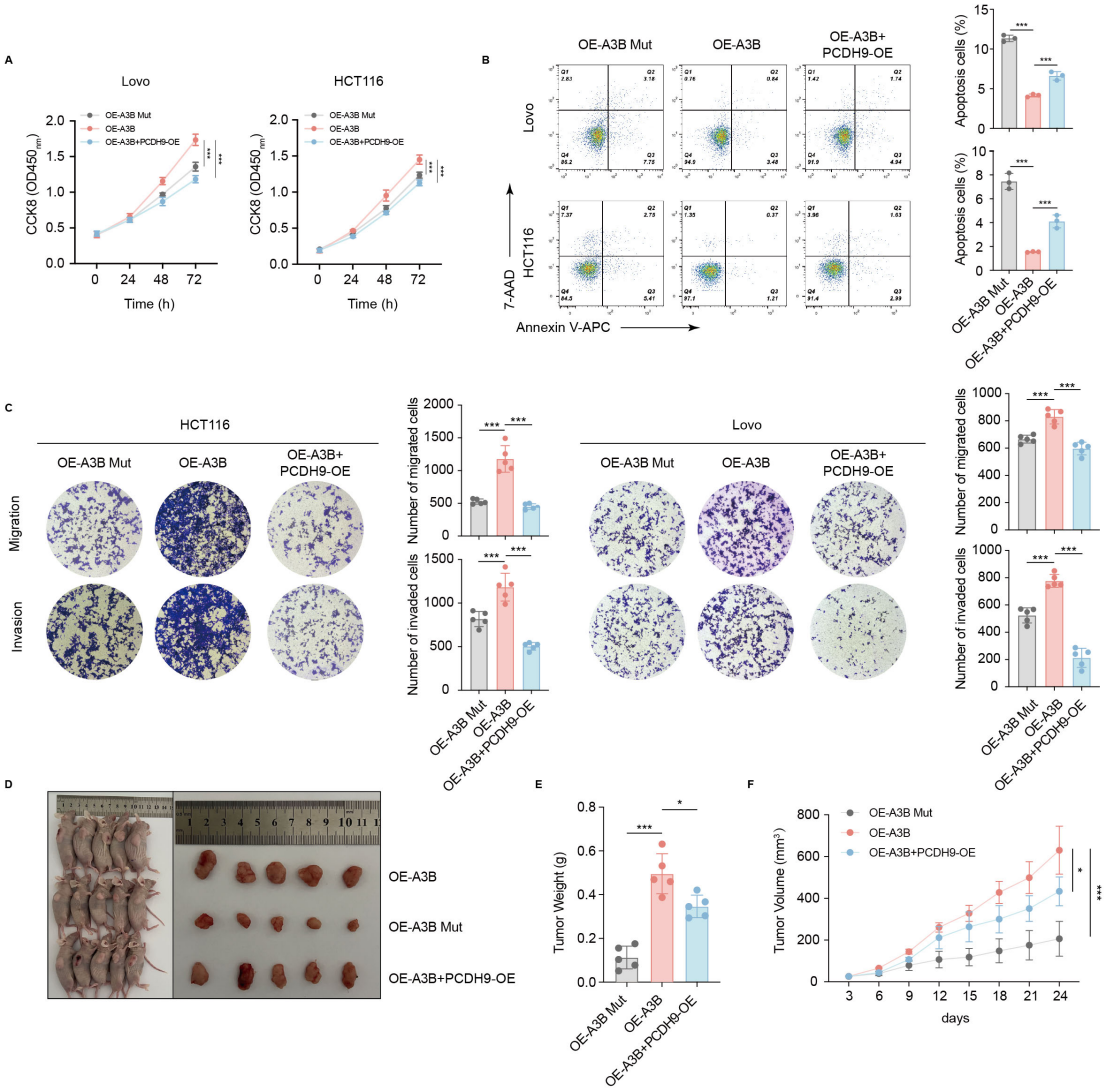
PCDH9 suppresses the malignant phenotypes of OE-APOBEC3B CRC cells *in vitro* and *in vivo*. **(a)** CCK-8 assay showing the effects of PCDH9 overexpression on proliferation of OE-A3B Mut, OE-A3B, and OE-A3B+PCDH9-OE CRC cells (LoVo and HCT116). **(b)** Flow-cytometry analysis of apoptosis in the indicated groups; bar plots quantify total apoptotic rates (early + late apoptosis). **(c)** Transwell migration (top) and invasion (bottom) assays showing that PCDH9 overexpression reduces motility and invasiveness of OE-A3B CRC cells; bar plots quantify migrated and invaded cell numbers. **(d)** Representative images of xenograft tumors derived from HCT116 cells transfected with OE-A3B Mut, OE-A3B, or OE-A3B+PCDH9-OE constructs (n = 5 per group). **(e)** Quantification of tumor weight at endpoint. **(f)** Tumor-growth curves showing tumor volume progression over time in the three groups. * p < 0.05, ** p < 0.01, *** p < 0.001, **** p < 0.0001.

Functionally, PCDH9 overexpression significantly inhibited proliferation, increased apoptosis, and reduced migration and invasion in OE-A3B CRC cells (**Figures 8a–c**). *In vivo*, PCDH9 restoration effectively neutralized the tumor-promoting effects of APOBEC3B activation, resulting in substantially smaller tumors in xenograft models (**Figures 8d–f**).

Together, these results identify PCDH9 as a candidate effector in APOBEC-associated tumor-promoting signaling, supporting a role for PCDH9 restoration and reversal of APOBEC3B-associated signaling changes, downstream pathway activation, and enhanced malignant phenotypes in CRC.

## 4 Discussion

CRC exhibits substantial heterogeneity in evolutionary trajectories and TME interactions, yet the correlates of this complexity remain incompletely understood. In this study, we identify AAS as a major feature of an aggressive CRC subtype characterized by transcriptional remodeling, stromal enrichment, altered vascular states, and PCDH9-linked signaling alterations. APOBEC was associated with expansion of immunosuppressive stromal populations, enrichment of arterial-like and pro-angiogenic endothelial states, and disruption of intracellular tumor suppressive signaling through PCDH9. Together, these findings support a model in which APOBEC-associated mutational and transcriptional programs are linked to aggressive CRC ecosystems characterized by pro-angiogenic and immunosuppressive features.

Although APOBEC-mediated mutagenesis is a well-established source of genomic diversification across cancer types, its biological and clinical relevance in CRC has remained only partially understood. Prior studies have mainly cataloged SBS2/13 signatures or linked APOBEC activity to hypermutation (22–31), without clarifying how this mutational process influences tumor microenvironment organization or lineage-specific cellular behavior. Emerging work in other cancers, such as EGFR-mutant lung cancer, has shown that APOBEC activity can promote cellular plasticity and therapy-resistant phenotypes, suggesting broader functional roles beyond mutation accumulation (32). In CRC, APOBEC-associated kataegis has been reported in immune-active tumors, yet existing analyses did not address whether APOBEC activation drives coordinated stromal expansion, rewires intercellular signaling, or shapes vascular specialization (33). Building on these observations, our integrated analyses suggest that APOBEC-associated mutational activity is linked to coordinated stromal, immune, and vascular alterations in CRC. Clinically, the resulting AAS stratified patient outcomes and was associated with advanced disease, supporting its potential value as a prognostic biomarker.

APOBEC family member specificity also warrants careful consideration. APOBEC3A and APOBEC3B are both capable of contributing to TCW-context mutagenesis, and mutational signatures such as SBS2 and SBS13 cannot by themselves definitively distinguish the responsible APOBEC enzyme. To address this issue, we compared APOBEC3A and APOBEC3B expression and evaluated their relative predictive association with TCW mutagenesis. APOBEC3B showed significantly higher expression than APOBEC3A in CRC tumors, and random forest analysis assigned greater predictive importance to APOBEC3B than to APOBEC3A for TCW mutation burden. These findings support the rationale for focusing the experimental validation on APOBEC3B. However, they do not exclude a contribution from APOBEC3A or other APOBEC family members. Therefore, we interpret AAS and patient-level TCW/SBS2/SBS13 features as APOBEC-associated signatures rather than strictly APOBEC3B-specific readouts. Future studies using APOBEC3A- and APOBEC3B-specific loss-of-function models will be required to determine their relative causal and functional contributions.

Mechanistically, our findings support a model in which APOBEC-associated mutational activity may contribute to transcriptional and microenvironmental alterations rather than representing a purely passive mutational byproduct. In response to the limitation of transient overexpression, we further established a doxycycline-inducible Tet-On APOBEC3B model and performed longitudinal WES across 0, 4, 8, and 12 weeks. This analysis showed that sustained APOBEC3B induction, but not doxycycline exposure alone, Tet-On background, or catalytically dead APOBEC3B, resulted in progressive TCW-context mutation accumulation. These results support the catalytic-activity-dependent and time-dependent nature of APOBEC3B-induced mutagenesis. Nevertheless, this experimental system remains an approximation of endogenous tumor evolution and cannot fully reproduce the chronic, heterogeneous, and selective pressures occurring in human CRC over years. In this model, APOBEC3B induction was accompanied by changes in secreted factors, including WNT7B and BMP6, suggesting a possible link between sustained APOBEC3B activity and tumor-cell secretome remodeling. These secretome alterations may contribute to stromal and endothelial transcriptional changes, although direct mutation-specific causal links remain to be established. Consistent with this possibility, AAS-enriched tumors showed increased ECM-remodeling, pro-angiogenic, and TGF-β/Wnt-β-catenin signaling programs. These findings extend prior observations that CAF-rich niches promote immunosuppression and angiogenesis in CRC, while suggesting that APOBEC-associated activity may be linked to such stromal states. At the same time, pan-cancer analyses have shown that APOBEC/AID mutational signatures correlate with distinct immune infiltration patterns, supporting our observation that high APOBEC-associated activity co-segregates with reduced anti-tumor immunity. Collectively, these results support a model in which APOBEC-associated mutational programs are linked to stromal–immune–vascular remodeling in CRC.

ECs are intrinsically plastic and can dynamically adjust to microenvironmental cues such as hypoxia, inflammation, growth factors, and matrix remodeling. This plasticity provides a biological basis for the endothelial state heterogeneity observed in CRC. In our study, AAS-enriched tumors showed increased vascular and angiogenic signatures, and single-cell analyses identified enrichment of endothelial populations with arterial-like and pro-angiogenic features. Importantly, the original Monocle2-based pseudotime inference was further supported by Monocle3 trajectory reconstruction and vector-based directional analysis, both of which indicated a consistent transcriptional continuum from venous/capillary-like endothelial states toward arterial-like endothelial states. These findings should be interpreted as convergent computational evidence for endothelial state directionality rather than definitive proof of developmental lineage conversion. The inferred arterial-like endothelial state was characterized by higher angiogenic activity, stress-response programs, and matrix-related signaling, features that may contribute to the pro-angiogenic and immunosuppressive vascular niche observed in AAS-enriched tumors. Clinically, higher arterial endothelial abundance was associated with poorer survival, suggesting that endothelial state remodeling may have prognostic relevance. However, whether APOBEC-associated tumor signals actively induce venous-to-arterial endothelial conversion *in vivo* remains to be tested by lineage-tracing and functional perturbation studies.

At the intracellular level, PCDH9 emerged as a candidate effector linked to APOBEC-associated signaling alterations. PCDH9 has been described as a context-dependent tumor suppressor in several malignancies, but its potential relationship with APOBEC-associated mutagenesis has not been well characterized (34). In our model, TCW-context mutations were detected within the cadherin repeat domain of PCDH9, and structural modeling predicted that these alterations may affect the extracellular domain conformation. These observations suggest a potential functional impact of PCDH9 alteration, but they do not by themselves demonstrate mutation-specific causality. Functionally, OE-A3B CRC cells showed reduced phosphorylation of MST1/2, LATS1, and YAP, together with activation of Wnt/β-catenin and TGF-β pathway markers. Restoration of PCDH9 attenuated these signaling changes and reduced malignant phenotypes *in vitro* and tumor growth *in vivo*. These findings support PCDH9 as a candidate component of the APOBEC-associated signaling alteration program. However, they do not establish that the specific PCDH9 TCW mutation is the causal event responsible for Hippo pathway suppression, nor do they exclude additional APOBEC-modified genes acting in parallel. In particular, mutant-specific PCDH9 rescue, CRISPR-based repair of the mutated TCW site, PCDH9 knockdown in OE-APOBEC3B Mut cells, and direct deamination assays using PCDH9 DNA fragments were not performed. Future studies using mutation-specific rescue and loss-of-function systems will be required to define the precise causal contribution of PCDH9 alteration to APOBEC-associated CRC progression.

The limited overlap between the patient-derived AAS and the OE-A3B cell model also warrants careful interpretation. In our OE-A3B model, 7 of the 22 AAS genes were significantly altered, indicating that the *in vitro* system does not fully reproduce the entire patient-derived signature. This is biologically plausible because AAS was constructed from bulk tumor cohorts and captures a composite transcriptional phenotype shaped by cancer cells, stromal and endothelial compartments, immune infiltration, and long-term tumor evolution. In contrast, the APOBEC3B model represents a simplified tumor-cell-intrinsic system. Therefore, we interpret the APOBEC3B-responsive genes as a cell-autonomous subset of the AAS that provides mechanistic leads, rather than as full experimental recapitulation of the entire signature.

Whereas current CRC biomarker development has focused largely on blood-based assays for early detection (35), our findings provide a complementary tissue-based, APOBEC-associated prognostic biomarker that stratifies CRC patients with distinct stromal, vascular, and immune features, suggesting potential value for prognosis evaluation and for generating hypotheses regarding anti-angiogenic therapeutic responses. In addition, PCDH9 may represent a candidate intracellular vulnerability within APOBEC-high tumors, although therapeutic relevance requires further functional and preclinical validation. Nevertheless, several limitations should be acknowledged. First, although multi-cohort bulk transcriptomic and single-cell analyses consistently linked AAS with stromal and endothelial remodeling, these findings remain primarily associative. The conditioned-medium experiments support a possible tumor-cell–secretome–TME axis, but they do not isolate the specific contribution of APOBEC-induced TCW mutations from other consequences of APOBEC3B activation. Second, although Monocle3 and vector-based analyses provided concordant computational support for a venous/capillary-to-arterial-like endothelial state continuum, these approaches infer directionality from transcriptomic state relationships and do not demonstrate *in vivo* endothelial lineage conversion. Third, AAS construction involved multi-step feature selection in TCGA-CRC, which may introduce optimism despite external validation; therefore, AAS should be regarded as an exploratory APOBEC-associated prognostic signature requiring prospective validation. Fourth, the OE-A3B model recapitulated only a subset of the patient-derived AAS genes, consistent with the composite tumor-level nature of AAS. Fifth, the structural modeling of mutant PCDH9 is based on computational prediction without experimental validation of protein stability or binding interactions. Sixth, although PCDH9 restoration attenuated APOBEC3B-associated signaling changes, mutation-specific causality remains unproven, and additional APOBEC-modified genes may act in parallel. Future studies using APOBEC loss-of-function models, mutation-specific PCDH9 rescue or repair systems, secretome-blocking assays, spatial transcriptomics, lineage tracing, co-immunoprecipitation, and prospective clinical cohorts will be required to validate the proposed model and assess its therapeutic relevance.

In summary, our revised interpretation positions APOBEC-associated activity as a marker and potential contributor to an aggressive CRC ecosystem characterized by stromal enrichment, arterial-like endothelial states, immune suppression, and PCDH9-linked tumorigenic signaling. While several mechanistic links remain to be experimentally validated, the integration of mutational, transcriptomic, single-cell, and functional evidence provides a framework for studying how APOBEC-associated processes intersect with tumor ecology and clinical outcome in CRC.

## 5 Conclusion

In conclusion, our multi-omics study identifies AAS that defines a CRC subtype characterized by stromal dominance, vascular specialization, and immune suppression. We further identify PCDH9 as a candidate gene associated with APOBEC-related signaling alterations, suggesting a potential link to the activation of tumorigenic Hippo, Wnt/β-catenin, and TGF-β pathways. These findings highlight the APOBEC-associated program as both a prognostic biomarker and a potential therapeutic vulnerability, offering opportunities for risk stratification and the development of tailored strategies in CRC.

## Data Availability

The raw data supporting the conclusions of this article will be made available by the authors, without undue reservation.
