# Supplementary material for: Association of APOBEC mutagenesis with stromal and endothelial niche remodeling and PCDH9-linked signaling alterations in colorectal cancer
Source: Front Immunol. 2026 Jul 16;17:1835351. doi: 10.3389/fimmu.2026.1835351 (PMC13422492; doi:10.3389/fimmu.2026.1835351)
Supplement: Supplementary file 1 [file Table1.docx]

## **
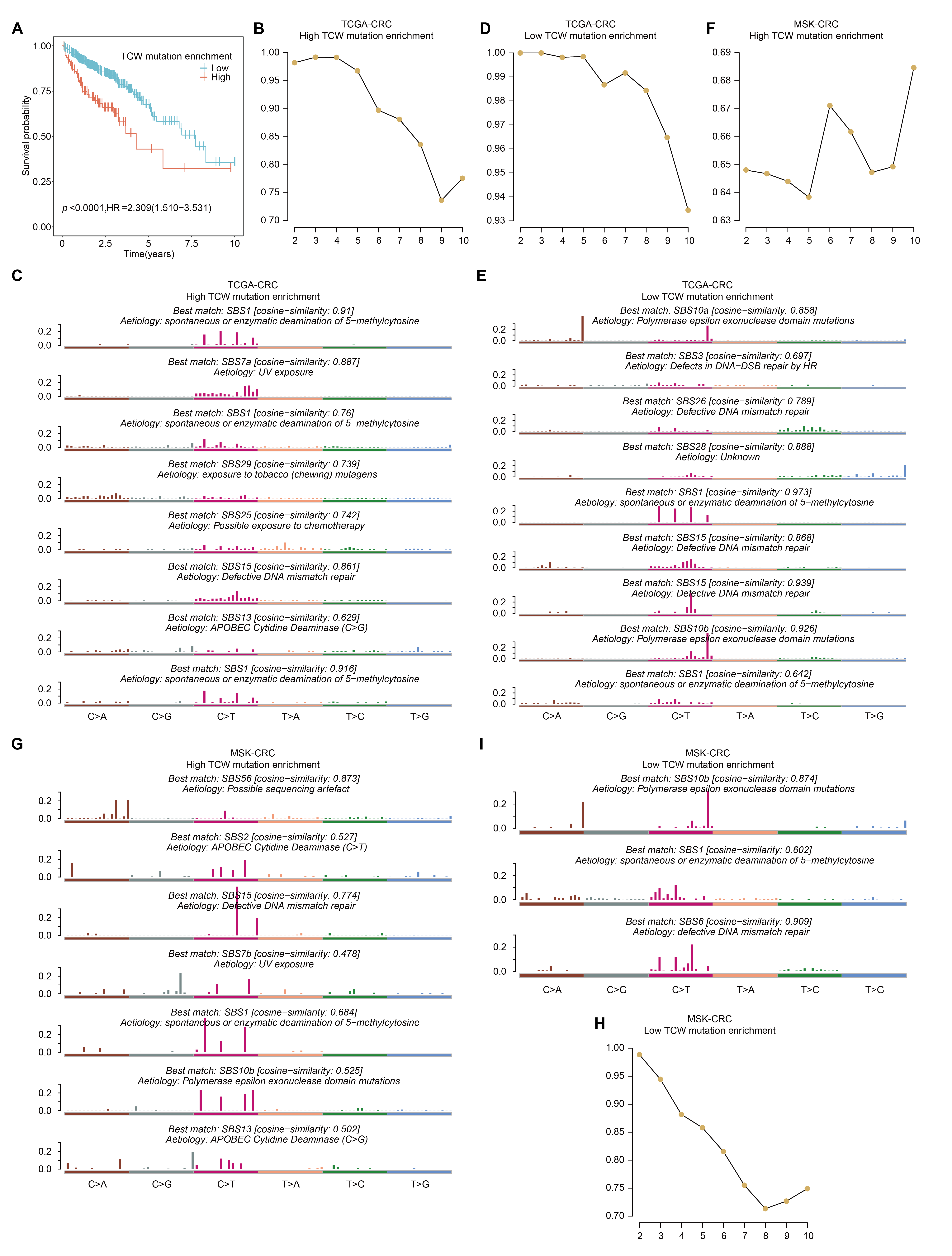
**

**Supplementary Figure 1.** **Comprehensive analysis of TCW mutation enrichment and mutational signatures in TCGA-CRC and MSK-CRC cohorts.** (A) Kaplan-Meier survival curves comparing patient outcomes stratified by high and low TCW mutation enrichment in TCGA-CRC cohort. (B) The cophenetic metric for high TCW mutation enrichment group in TCGA-CRC cohort was calculated across various cluster numbers, ranging from 2 to 10. (C) Mutational signatures identified in high TCW mutation enrichment group of TCGA-CRC cohort. (D) The cophenetic metric for low TCW mutation enrichment group in TCGA-CRC cohort was calculated across various cluster numbers, ranging from 2 to 10. (E) Mutational signatures identified in low TCW mutation enrichment group of TCGA-CRC cohort. (F) The cophenetic metric for high TCW mutation enrichment group in MSK-CRC cohort was calculated across various cluster numbers, ranging from 2 to 10. (G) Mutational signatures identified in high TCW mutation enrichment group of MSK-CRC cohort. (H) The cophenetic metric for low TCW mutation enrichment group in MSK-CRC cohort was calculated across various cluster numbers, ranging from 2 to 10. (I) Mutational signatures identified in low TCW mutation enrichment group of MSK-CRC cohort.


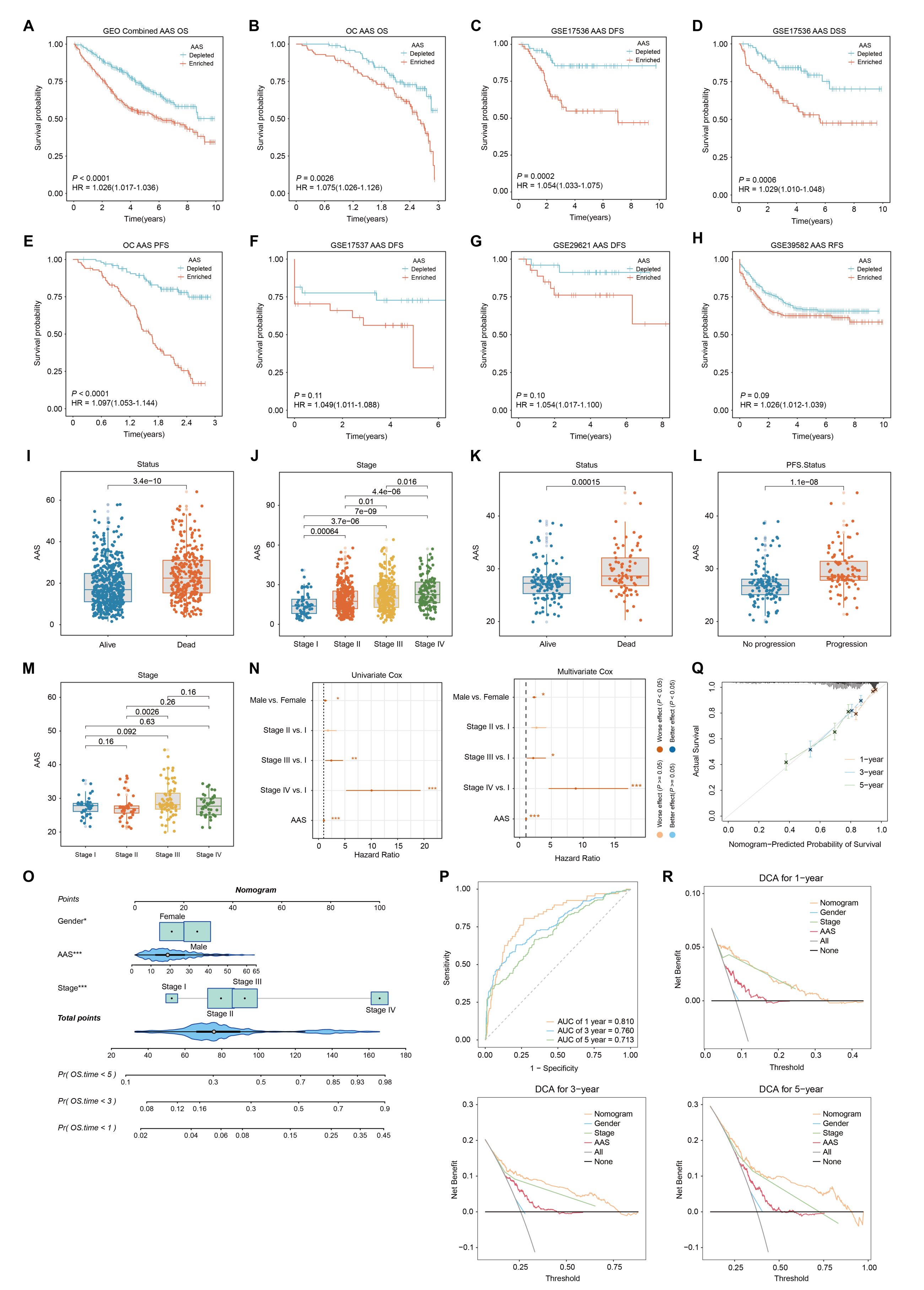


**Supplementary Figure 2.** **Model evaluation and construction of a prognostic nomogram.** (A-H) Kaplan–Meier survival curves showing the impact of AAS on prognosis across multiple cohorts. Patients were stratified into AAS-enriched and AAS-depleted groups: Overall survival in combined GEO cohort (A); Overall survival in OC (B); Disease-free survival in the GSE17536 cohort (C); Disease-specific survival in the GSE17536 cohort (D); Progression-free survival in OC (E); Disease-free survival in the GSE17537 cohort (F); Disease-free survival in the GSE29621 cohort (G); Recurrence-free survival in the GSE39582 cohort (H). (I-M) Distribution of AAS in different clinical features across combined GEO cohort and OC. Comparisons were made by survival status, progression status, and clinical stage. (N) Univariate and multivariate Cox regression analyses of overall survival in combined GEO cohort. (O) Nomogram integrating gender, clinical stage, and AAS to predict 1-, 3-, and 5-year overall survival probabilities in combined GEO cohort. (P) ROC curves of the nomogram for 1-, 3-, and 5-year survival prediction. (Q) Calibration curves comparing nomogram-predicted survival probabilities with actual survival at 1, 3, and 5 years. (R) DCA assessing the clinical net benefit of the nomogram and individual predictors (gender, stage, AAS) for 1-year, 3-year, and 5-year survival. **p* < 0.05, ***p* < 0.01, ****p* < 0.001; OC, our CRC patient cohort.


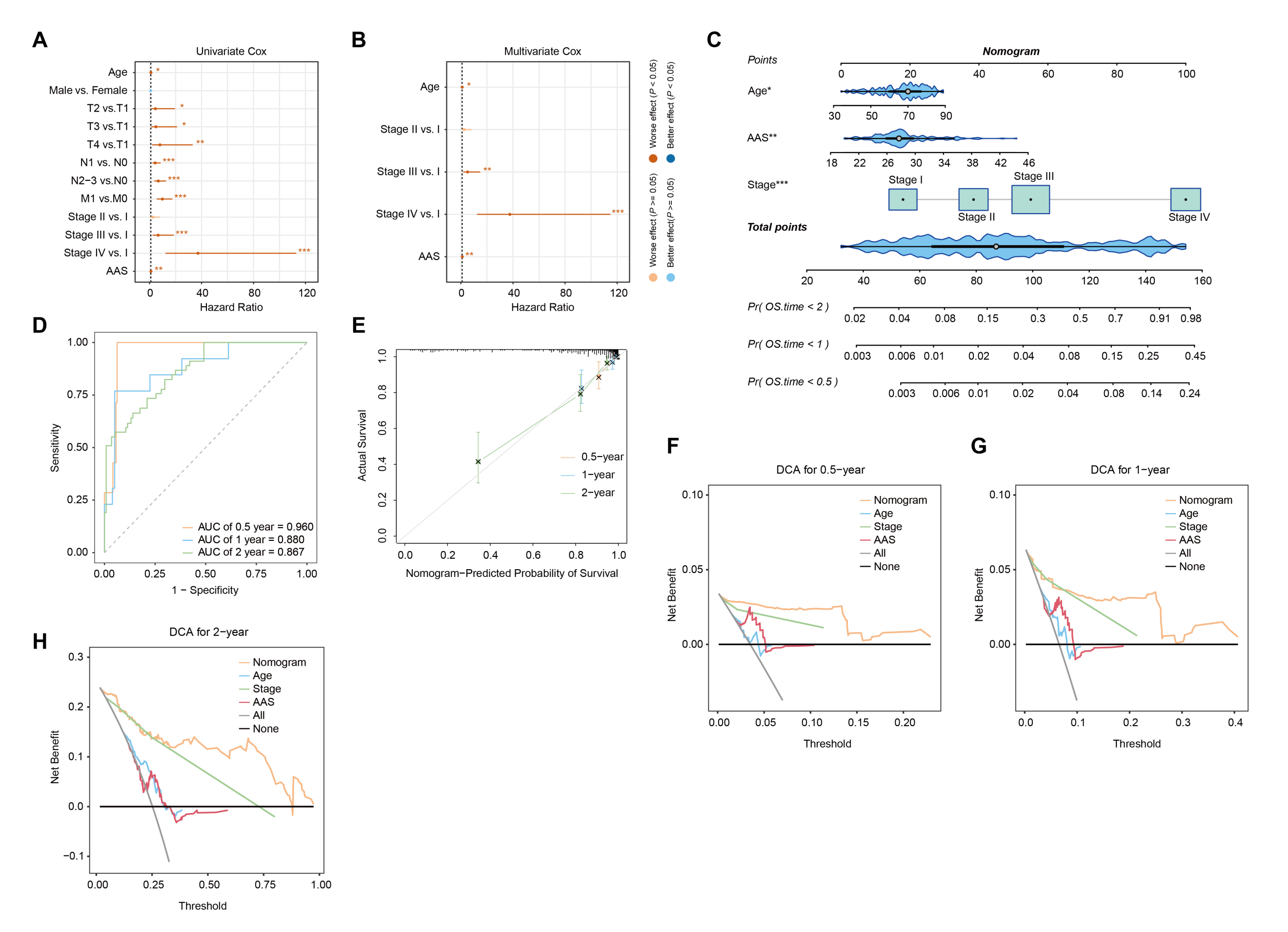


**Supplementary Figure 3.** **Model evaluation and construction of a prognostic nomogram.** (A-B) Univariate and multivariate Cox regression analyses of overall survival in OC. (C) Nomogram integrating age, clinical stage, and AAS to predict 0.5-, 1-, and 2-year overall survival probabilities in OC. (D) ROC curves of the nomogram for 0.5-, 1-, and 2-year survival prediction. (E) Calibration curves comparing nomogram-predicted survival probabilities with actual survival at 0.5, 1, and 2 years. (F-H) DCA assessing the clinical net benefit of the nomogram and individual predictors (age, stage, AAS) for 0.5-year, 1-year, and 2-year survival. **p* < 0.05, ***p* < 0.01, ****p* < 0.001; OC, our CRC patient cohort.


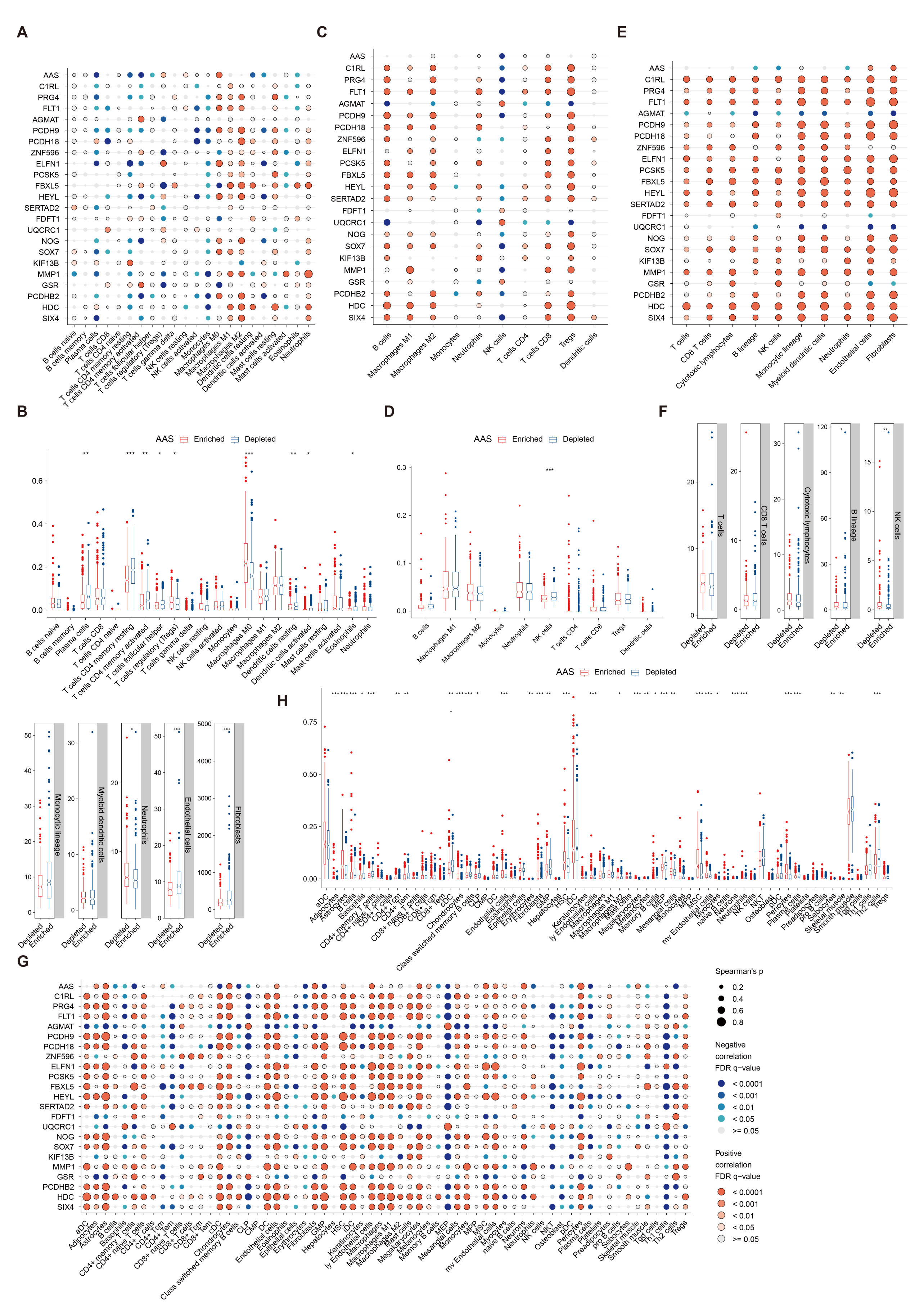


**Supplementary Figure 4.** **Association between immune-stromal cell infiltration and AAS.** (A-H) Analysis of immune and stromal cell infiltration using CIBERSORT, quanTIseq, MCP-counter, and xCell: (A, C, E, G) Correlations between cell type abundance and AAS or its component genes. (B, D F, H) Comparisons of cell type abundance between AAS-enriched and AAS-depleted groups. **p* < 0.05, ***p* < 0.01, ****p* < 0.001, *****p* < 0.0001.


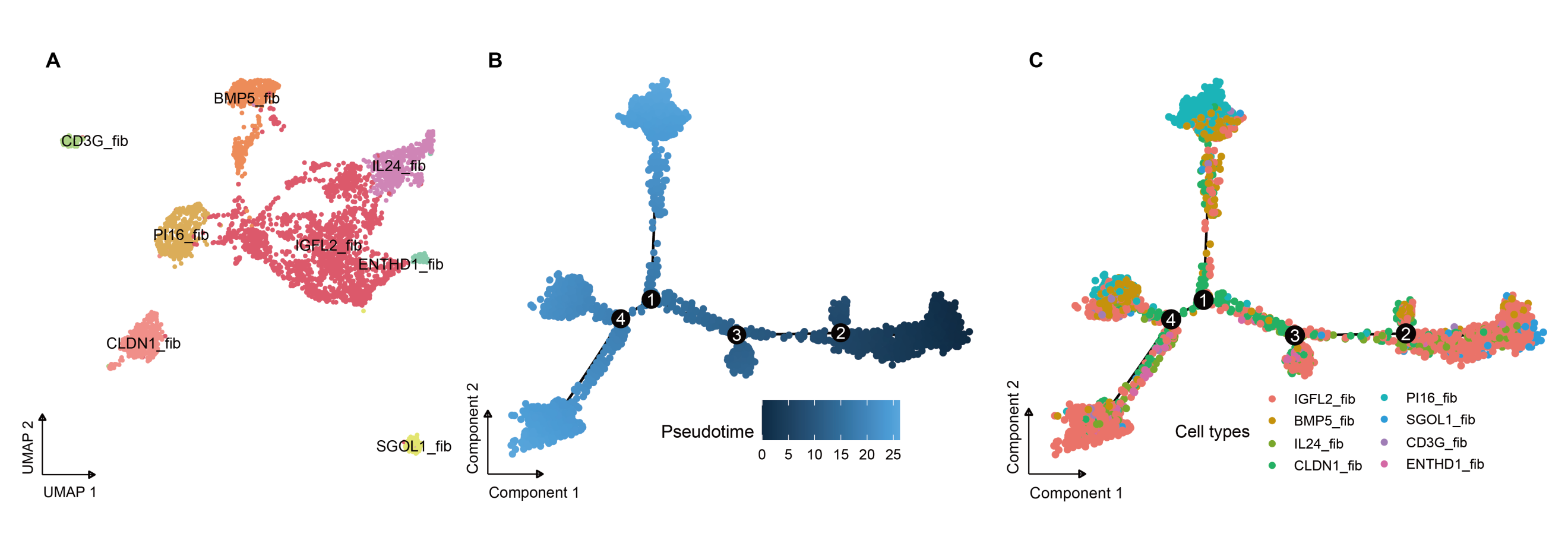


**Supplementary Figure 5.** **Characterization of fibroblast populations and developmental trajectories.** (A) UMAP plot shows the clustering and annotation of distinct fibroblast populations. (B) Trajectory analysis illustrating the inferred developmental progression of fibroblasts along the pseudotime. (C) Fibroblast subtypes mapped onto the same trajectory, showing their distribution along the differentiation path.


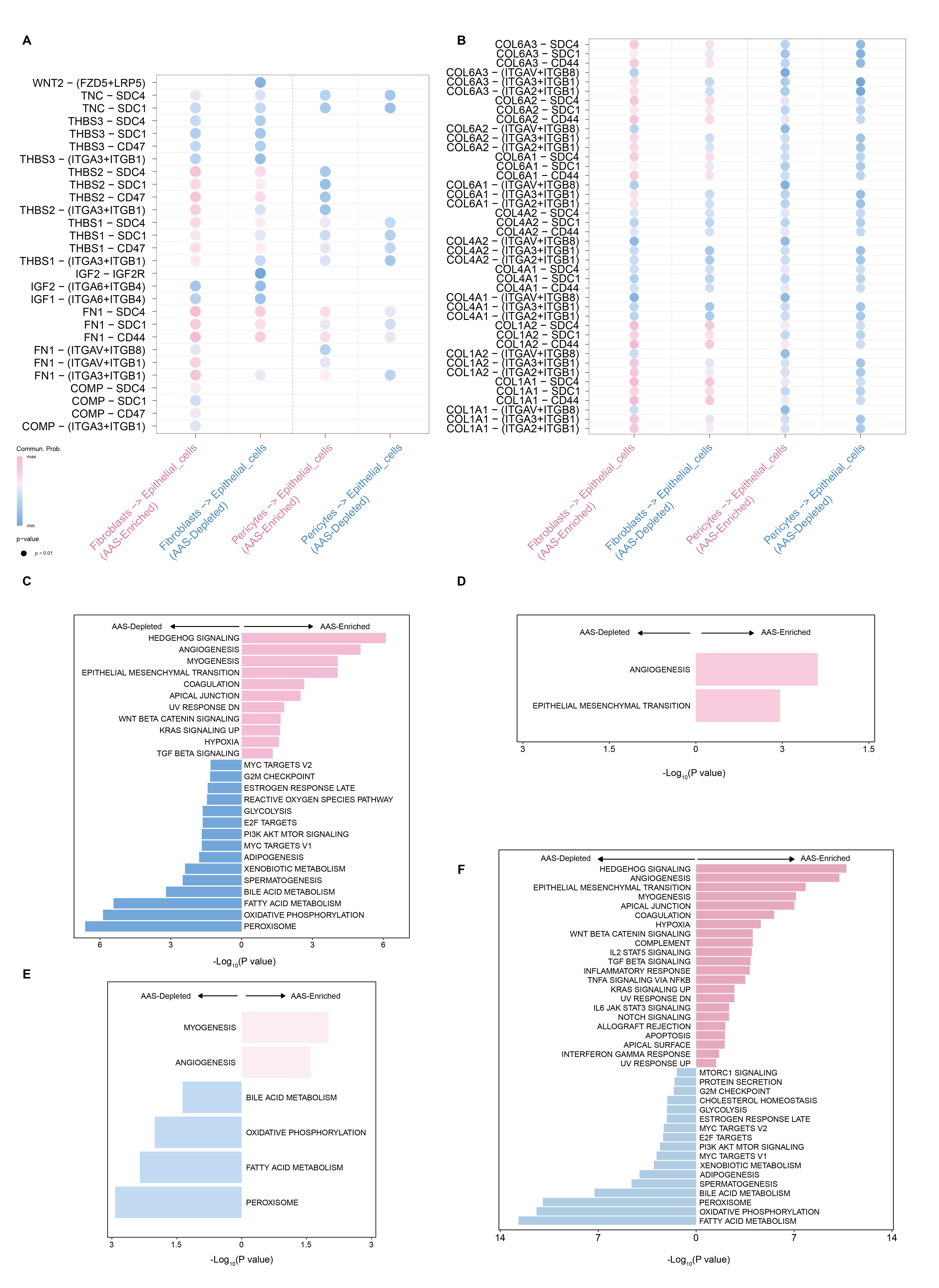


**Supplementary Figure 6.** **Intercellular communication and functional pathway differences between AAS-enriched and AAS-depleted groups across multiple cohorts.** (A-B) Bubble plot illustrating significant ligand-receptor pairs mediating intercellular communication between specific sender and receiver cell types in AAS-enriched and AAS-depleted groups. (C-F) Bar plot representing significantly enriched biological functions in AAS-enriched and AAS-depleted groups in four cohorts: GSE17536 (C); GSE17537(D); GSE29621 (E); GSE39582 (F).


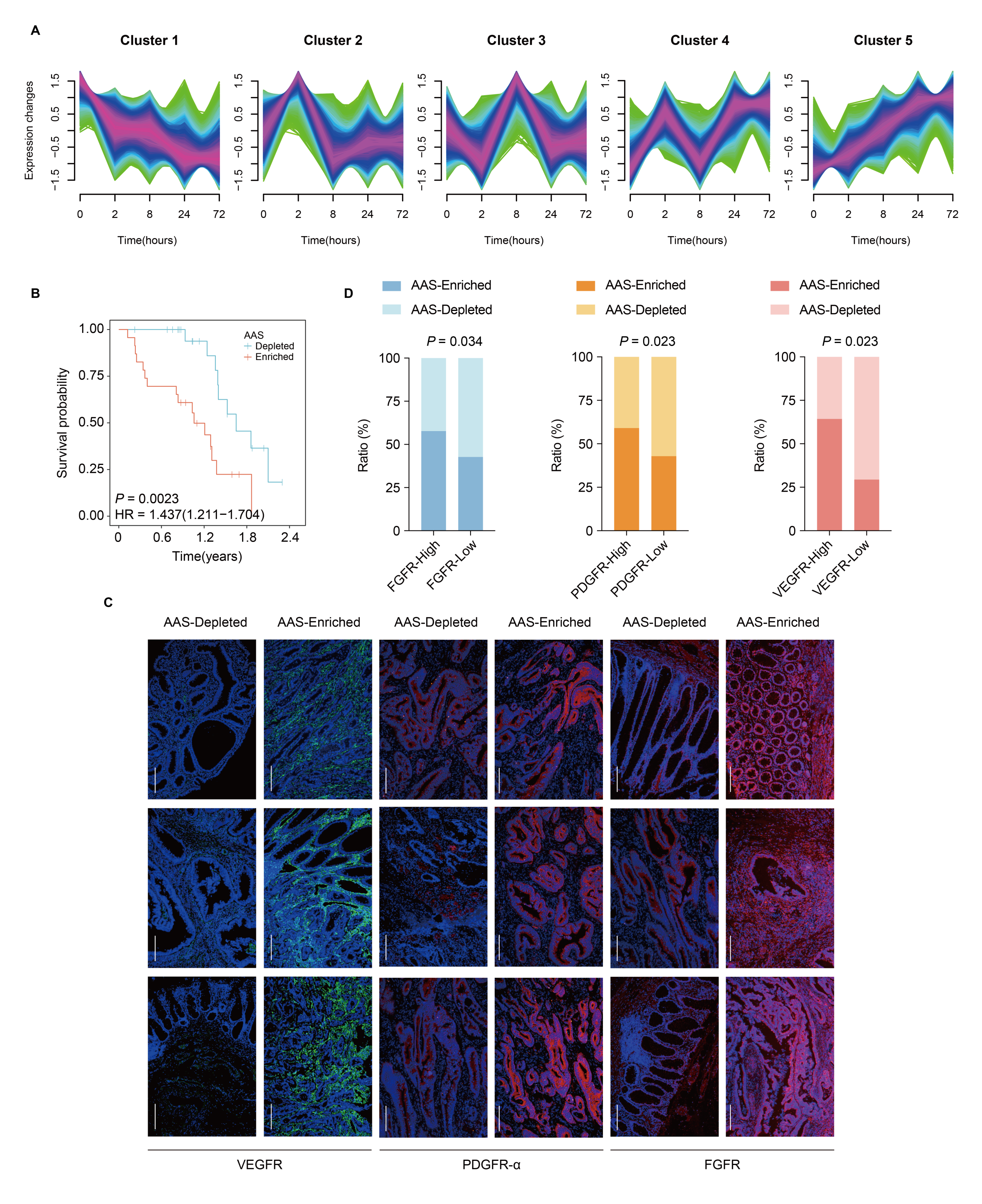


**Supplementary Figure 7.** **Association of AAS with tumor angiogenesis and patient survival.** (A) Cluster analysis by the Mfuzz identifying five clusters of temporal expression changes trajectories. (B) Kaplan-Meier overall survival curves for patients receiving anti-angiogenic agents, comparing those with AAS-enriched versus AAS-depleted group. (C) Representative immunofluorescence images show VEGFR (green), PDGFR-α (red) and FGFR (red) expression patterns in tumor tissues. Nuclei are counterstained with DAPI (blue). Scale bar = 200 µm. (D) Proportions of AAS-enriched and AAS-depleted groups stratified by VEGFR expression (left), PDGFR-α expression (middle), and FGFR expression (right).


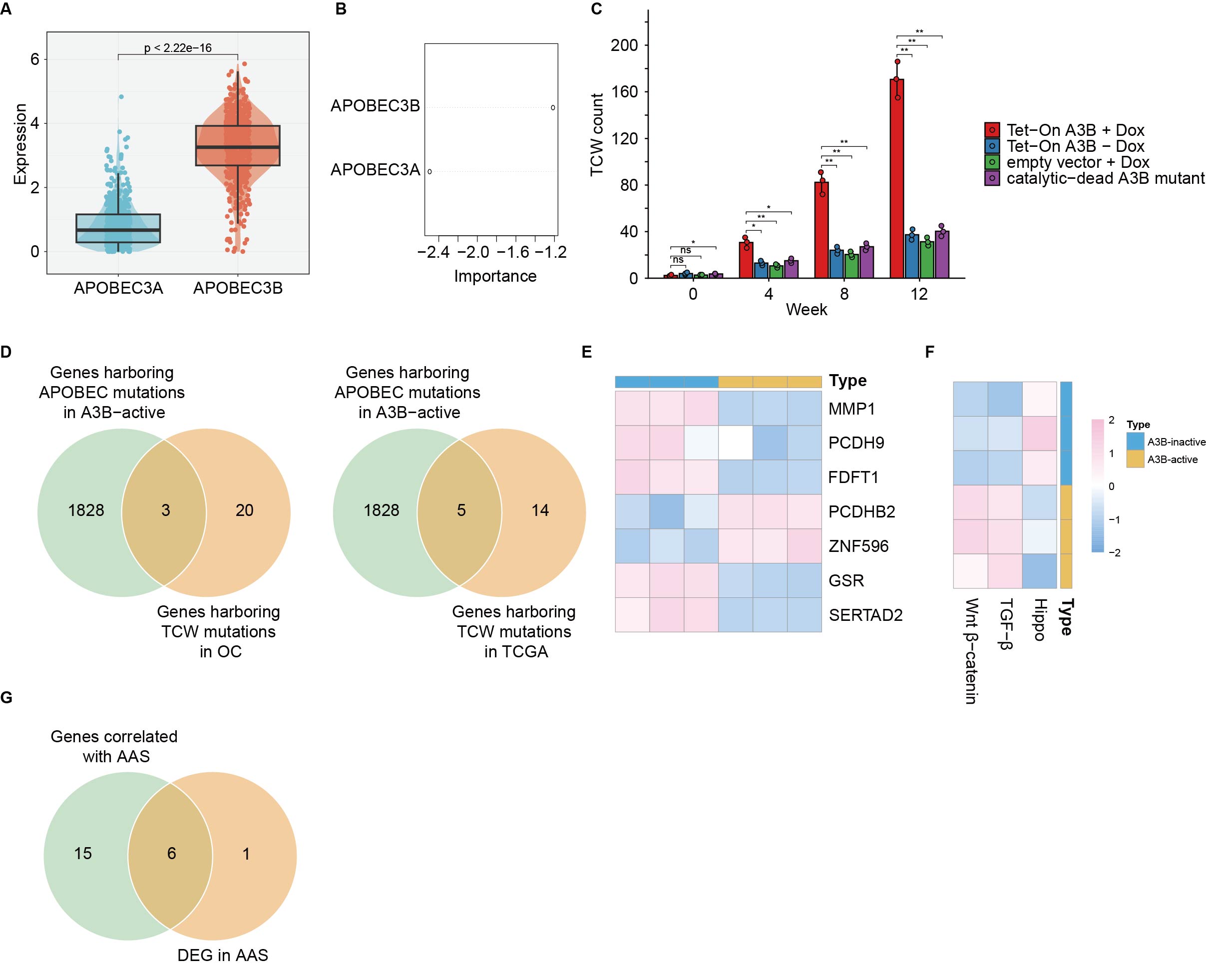


**Supplementary Figure 8.** **Expression of 22 genes and pathway alterations in OE-APOBEC3B CRC cells.** (A) Comparison of the expression levels of APOBEC3A and APOBEC3B in the TCGA cohort. (B) Variable importance was estimated using random forest. (C) Quantification of TCW-context mutations detected by WES across the four groups and four time points. (D) Venn diagram showing the overlap between the top 20% of genes with TCW mutations identified by WES in OE-APOBEC3B CRC cells and the high-frequency mutated genes (>2%) detected in our own cohort (left) and TCGA cohort (right). (E) Heatmap of the expression profiles of the 7 significantly differentially expressed genes between OE-APOBEC3B and OE-APOBEC3B Mut cells. (F) Heatmap illustrating the activity scores of the Hippo, Wnt/β-catenin, and TGF-β signaling pathways in OE-APOBEC3B and OE-APOBEC3B Mut groups. (G) Venn diagram showing the overlap between significantly differentially expressed genes and those significantly correlated with AAS.
